# A 4D hyperchaotic image encryption scheme using Circular Radius–Angle Transformation and bit-level permutation

**DOI:** 10.1371/journal.pone.0353752

**Published:** 2026-08-28

**Authors:** Mohammed Abdullah Khalaf, Usman Ullah Sheikh, Shahidatul Sadiah Binti Abdul Manan, Sabah Fadhel Hamood, Najamuddin

**Affiliations:** 1 Faculty of Electrical Engineering, Universiti Teknologi Malaysia (UTM), Johor Bahru, Johor, Malaysia; 2 Scientific Research Commission, Baghdad, Iraq; 3 Middle Technical University- Technical Institute Balad, Salah Al-Din, Iraq; Jaramogi Oginga Odinga University of Science and Technology, KENYA

## Abstract

With the rapid growth of digital data transmission, secure image encryption has become a critical requirement for protecting visual information against unauthorized access and cryptanalytic attacks. Existing chaotic-based encryption schemes often suffer from limited resistance to statistical and differential attacks due to weak key dependence, low-dimensional chaotic behavior, or the partial use of image features during encryption. To overcome these limitations, this study proposes a novel four-dimensional (4D) hyperchaotic image encryption mechanism that is independent of image statistical features and semantic information, thereby making cryptanalysis highly infeasible. The proposed algorithm utilizes a 4D Lorenz hyperchaotic system to generate the primary pseudo-random key sequences, which are then processed using mathematical and modular operations to produce dynamic subkeys for each encryption stage. The encryption process consists of three structured phases: (1) initial pixel sorting based on the first and second keys; (2) Circular Radius–Angle Transformation (CRAT**)** using the third and fourth keys to remap pixel positions in a nonlinear circular coordinate space; and (3) a final diffusion phase using the fifth key to perform XOR-based confusion. Experimental evaluations confirm that the proposed approach achieves high key sensitivity, a vast key space, and strong resistance to statistical, differential, and chosen-plaintext attacks. Furthermore, the encrypted images demonstrate uniform histograms, minimal pixel correlation, high information entropy, and excellent computational efficiency, highlighting the algorithm’s robustness and applicability for secure image transmission in real-world scenarios.

## 1 Introduction

With the significant advances in computer networks and the continuous storage and transmission of digital data, consumers have become increasingly aware of the substantial privacy risks, and the confidentiality of information has become a crucial issue [1]. In multimedia communication, the secure storage and transmission of digital images constitute a key challenge [2]. To protect digital data from unwanted access and criminal activity, there are three methods: cryptography, steganography, and watermarking [3]. The significant role of the cryptography system is to offer robust and secure transmission over an unconfident channel. Cryptographic techniques are divided into types: block and stream ciphers. Stream ciphers encrypt digital data bit by bit using a secret key, whereas block ciphers encrypt whole blocks of bits. Stream ciphers are commonly used in RC4 and (LFSR) linear feedback shift registers. The block ciphers include the Advanced Encryption Standard (AES) algorithm, the Data Encryption Standard (DES) algorithm, and triple DES (3DES) algorithm [4]. Because image data needs strong real-time properties in communications, traditional encryption techniques are unsuitable for image encryption [5]. Furthermore, for real-time image encryption, these ciphers demand greater processing time, computing resources, and consume significant power [6]. Moreover, several fundamental characteristics of images, such as high data redundancy, huge storage capacity, and significant correlation between neighboring pixels, distinguish them from other types of information [7]. According to Shannon’s concept, high-level security can be achieved by incorporating two essential and complementary stages: confusion, which obscures the relationship between plaintext and ciphertext, and diffusion, which spreads the influence of each bit over many ciphertext bits,a confusion level utilizes many parts of the key to force each bit in an image during encryption, thus obliterating the relation between the two,a diffusion level has an avalanche impact, where changing one of the bits in the plain image can change almost half of all bits in the ciphered image, a diffusion level utilize to obfuscates the statistical information between the original and a ciphered image [8,9]. Hyper chaotic cryptography possesses several inherent characteristics that make it particularly well-suited for image encryption. These systems exhibit extreme sensitivity to initial conditions, non-periodicity, ergodicity, and strong pseudo-randomness, enabling the generation of complex and unpredictable hyper chaotic sequences with high reliability [10]. Unlike low-dimensional chaotic systems, hyper chaotic systems operate in higher dimensions and are characterized by multiple positive Lyapunov exponents, which enhance the complexity of their dynamic behavior and produce superior randomness for cryptographic operations [11]. This multi-dimensional nature allows for the construction of dynamic permutation and diffusion mechanisms that strengthen resistance to statistical and differential attacks [12]. Moreover, the vast key space generated by hyper chaotic systems provides additional protection against brute-force and chosen-plaintext attacks [13]. In general, chaotic cryptosystems can be categorized as either single-dimensional or multi-dimensional. While single-dimensional chaotic maps are easier to implement and efficient for real-time encryption applications, multi-dimensional and hyperchaotic maps achieve stronger diffusion and confusion properties at the cost of greater algorithmic complexity.

Most of the literature review provided algorithms that utilize more than a single or multi-dimensional chaotic map to carry out the high levels of cryptosystem of images. In [14], the author applied a merged operation between the logistic map and Chen system and then utilized a zigzag technique for shuffling based on the dimensions of the original image to be ciphered. This approach enhances pixel correlation disruption through bidirectional crossover. The hyperchaotic sequences from the Chen system perform reversible diffusion. The researcher in [15], proposes a novel extended Zigzag transform that produces a great number of Zigzag-like routes. In addition, there is a complementary rule of eight-base DNA computing, the number of which is 5040 EDCREZT is a novel hyperchaotic encryption algorithm for color images that merges complementary eight-base DNA rules, a (4D) hyperchaotic system, and the newly advanced extended Zigzag transform. The EDCREZT encryption includes five stages that the plain image will go through. First, DNA-stage permutation through the new expanded pixel-level permutation; second, dynamic encoding for DNA; third, zigzag transformation; and then, dynamic decoding of DNA. Finally, the DNA diffusion stage uses two rounds. Then the cipher image is produced. The researchers in [16] utilized the approach of a dynamical key, combining elliptic curves with fraction-order chaotic maps. The algorithm confirms a reliable key exchange and hides transmitted data. The analysis of security is applied to performance and attacks. In [17], the author utilized the chaotic blockchain encryption for an image splitting security-sharing mechanism mainly includes a fine grained access control method based on Attribute Based Access Control (ABAC) and an image specific chaotic encryption scheme. The proposed fine grained access control method employs smart contracts based on the ABAC model to achieve automatic access control for private areas. It employs a Cuckoo filter based transaction retrieval technique to enhance the efficiency of smart contracts in retrieving security attributes and policies on the blockchain. In Reference [18], the author utilizes four fundamental chaotic maps that are united in pairs and connected with sine operations to form the basis of the author’s proposed image encryption algorithm. First, a six component sinusoidal chaos map (CSCM) was acquired in one dimension. Second, specify LCS and SCS, the two excellent chaos mappings. Thirdly, it simultaneously diffused and scrambled each pixel of the image during the encryption process, which is based on the shape of a traditional Chinese fan. This approach improves the speed of encryption significantly. When altering one pixel’s value, that might impact many succeeding pixels’ values in the diffusing stage. In the scrambling sequence, each pixel moves in relation to the three pixels in front of it. Reference [19] describes a double image ciphering technique through Industrial Internet of Things. The proposed technique enciphers two images into one color cipher text image; as a result, a high confusion for the analyst who tries to illegally break the cipher text image. Firstly, convert two images of size N × M into a 3D bit-level matrix of size N × M × 48. Next, the place of the units permutated from the resulting 3D bit-level matrix 3D non equilateral Arnold map (3D-NEAT) is utilized. Then, the permutated matrix is modified into three 2-D pixel-level images, and then random diffusion sequences are applied by the 3D Lorenz algorithm (3D-LS). Lastly, three diffused 2D pixel levels are scrambled using matrices generated by 3D-LS, and the result is three color components of the ciphered image. The work presented in [20], the authors proposed a technique based on three stages. The first level generates the first encryption key utilizing Rule 30 cellular automata. The second level used a well tested S-box, which is responsible for modular inverses, transformation, and permutation. Finally, the third level generates the second encryption key, utilizing the Lorenz system. The concept of confusion and diffusion of Shannon’s properties of a cryptographic system is applied in this technique. In Reference [21], the authors proposed an image encryption scheme that introduces a two – dimensional quadratic sine map (2D-QSM) to enhance the chaotic characteristics and overcome the limitations of conventional one-dimensional and low-dimensional chaotic maps. To establish a secure and dynamic key generation mechanism, the algorithm converts segmented image data into the initial parameters of the 2D-QSM. Additionally, a hash-based key derivation process is incorporated to strengthen the relationship between the encryption key and the plaintext. The encryption methodology further applies cyclic shifting and segmentation–recombination operations across the RGB channels. Researcher in [22], the authors proposed an image encryption scheme that integrates two chaotic systems Arnold’s Cat Map and the two-dimensional Logistic-Sine-Coupling Map (2D-LSCM)—to enhance randomness and strengthen encryption security. The methodology leverages the deterministic yet highly unpredictable nature of chaotic systems to ensure secure image storage and transmission. This scheme introduces chaotic randomness at multiple stages of the encryption process to improve diffusion and confusion properties. The researcher in [1] introduces a novel encryption technique based on the column-row scrambling technique. First, a 2D scrambled image is generated from an input image. Second, from the input image, the columns are randomly placed into the scrambled image columns. The process is reiterated to enhance the scrambling effect once all columns have been included. A parallel method is employed on the image for the row. Finally, for the diffusion stage, the XOR operation is used between the random number generated and the scrambled image to improve security. As explained in [23], the authors introduce an innovative shuffling approach that rearranges the pixels of a color image using the logistic map and Arnold’s cat map for the purpose of color image encryption. The first step of the technique is the shuffling by using a chaotic map to fulfil the required shuffling outcome. The plain image and image size are accepted as input. The pixel values of an image are stored in a 2D array, and another 2D array is generated to fill using a shuffling technique to produce a different value with each iteration. The indices of row and column values are calculated based on the 2D array values that were previously calculated, and the plain value of the pixel is shuffled with the new index values for row and column. In [24], the author provides the hyper-chaos map and a vector process. He introduced a high-processing mechanism to achieve a key array that decreases the number of iterations required for the hyperchaotic algorithm. The application of chaotic maps in encryption is limited when there is a small key space, and the security and performance are affected by low linear complexity. In addition, low periodic orbits of image encryption limit the effectiveness of encryption. The researchers in [25], employed a 3-phase approach to encrypt an image; they used a piece-wise linear chaos algorithm, logistic maps, and a chaos map that depends on the S-box. The PWLCM has a high positive Lyapunov exponent, which makes the algorithm extremely robust. The proposed scheme may have certain conditions that are weak under position sensitivity. Through parallel computing, the encryption scheme needs further evaluation of efficiency. In reference [26], the research suggested a method by combining many images of a satellite into an augmented image, which improved the security to resist traffic analysis. The encryption procedure separates the color image into three channels, RGB, and each channel goes through four levels. First level, the confusion is achieved by utilizing a hyperchaotic key transformed by singular value decomposition, and XOR operations with RC5 algorithm in counter mode, then using a 6D hyperchaotic key for Hill cipher encryption, invertible matrices mod 256 and utilizing modified Blum Blum Shub to generate S-box for substitution operation. As explained in [27], the authors introduce a new technique for ciphering images, where the first stage is combining many images into one image by merging the RGB channels, and the second stage is a 3D chaotic map generated for permutation based on the suggested S-box, and then the AES substitution method is used for substitution. In [28], the authors proposed a novel encryption scheme for medical images that integrates integer wavelet transform (IWT) with DNA encoding. The pseudo-random sequences are generated by utilizing a 3D hyperchaotic map. First, IWT is implemented to extract the approximation components of the images, and secondly, a novel diffusion algorithm masks critical information. Thirdly, rearranging pixel positions using a bit-level permutation mechanism enhances encryption complexity. Finally, a random DNA operation is used to encode the permuted images, and specialized DNA cubes are used to shuffle the DNA. In [29], the authors introduce a framework for image encryption based on the principles of chaos theory by using a cellular automata neighborhood (CAN) and a two-dimensional hyperchaotic map (2D-SGHM) derived from the Gauss and classic sine maps. The study is based on the basic performance and dynamic behaviors of this map. This study employs three encryption algorithms of image found from Moore neighborhoods, called OIEA, NIEA, and MIEA, and one-dimensional, von Neumann. In [30], the authors proposed a technique integrating memristors into a Hopfield neural network (HNN) to generate a range of dynamical behavior, which has effective implications for modeling and biomimetic applications of artificial neurons. The synapse of the second neuron is replaced by the proposed memristor to construct a memristive tri-neuron Hopfield neural network (MTN-HNN). As a result, a diverse range of dynamic behaviors can be generated from the outcomes, where the dynamics of the MTNHNN are inﬂuenced by the internal parameters of the memristor to extend attractors in up to two directions. In [31], the authors introduced medical image encryption scheme with high performance that combine a differentiated encryption technique (MIES-FHNN-DE). With a novel 4D fractional-order HNN. The MIES-FHNN-DE 4D fractional-order HNN generates keystreams by utilizing a 2D hyperchaotic map. The MIES-FHNN-DE enhancement encryption efficacy by pixel bit splitting and weighted gathering. In reference [32], the authors propose a logistic modular map (2D-ELMM) and an image encryption based on a chaotic scheme using a vector-level procedure and 2D-ELMM (CIES-DVEM).The secret key is applied to different images, and there is no need to recreate the sequence of chaotic in the encryption procedure. The dynamic of CIES-DVEM is widely correlated to plaintext images. In [33], the authors proposed a method that integrates a novel multi-dimensional hyperchaotic map with DNA cube-based operations to secure medical images in healthcare IoT environments. In this approach, the hyperchaotic system generates highly random key sequences used to guide DNA encoding, cube rotation, and dynamic rule-based substitution for pixel permutation and diffusion. The bits are then randomly inserted into images by a matrix convolution process. In reference [34] authors proposed a 3-phase image cryptosystem. In the first phase, they utilized a Tan difference of the logistic map to achieve (DNA) deoxyribonucleic acid encoding. For the next phase, the Lorenz system numerical solution and the algorithm of a linear descent are combined to generate an efficient S-box. In the third phase, the logistic map is applied. Through the first and third, the diffusion is applied. In the second stage, the implementation of the S-box achieves confusion [35]. In this research work, the authors utilized the Chen hyperchaotic system and extended it to a 4D fractional-order domain. The scheme included 3 stages. First, the discrete Fourier transform of the numerical solution of the fractional-order Chen system is applied and utilized for DNA coding. For the second stage, the DFT-quantized solution of the Chen system was applied to construct a robust S-box. Finally, to convert to base-φ, the encryption key of Mersenne Twister is utilized, and modulo operation is used.

In general, some methods have relied on one-dimensional chaotic methods such as logistic maps, Sine map and Tent map, and others have used two-dimensional chaotic algorithms such as Henon Map, Arnold’s Cat Map, Baker Map, and 3Dimensional or more, such as Lorenz, Chen, Rӧssler and Lȕ system while some authors have turned to methods that combine these two. Although the proposed image encryption algorithms show promising features, they still face several challenges. Limitations, such as restricted key space, low sensitivity to key changes, inconsistent performance under certain conditions, and vulnerability to statistical attacks, suggest that there is room for improvement. Future research should aim to strengthen key management, expand the complexity of the key space and technique, and ensure reliable performance across a wide range of applications in real time.

In addition to the previously developed four-dimensional Lorenz-type chaotic systems, which have been widely applied in image encryption, this work introduces a new encryption architecture. Here, the primary novelty lies in its encryption architecture, which strategically utilizes the sequences generated by these systems rather than proposing a new chaotic model. In reference [36], the authors introduce a hyperchaotic image encryption algorithm that combines a newly developed four-dimensional hyperchaotic system with DNA-based dynamic encoding. The strong pseudo-randomness of the chaotic system enables the generation of highly unpredictable control sequences that drive dynamic DNA encoding, calculation, and decoding throughout the encryption process. A plaintext-dependent keystream and a self-adapting permutation strategy—applied at both the bit level and DNA level. In [37] the authors present an image encryption method that integrates a hyperchaotic system with an enhanced bit-level permutation approach. The process begins with a forward diffusion process that uses two-dimensional XOR operations. Then, the scrambled image is permuted in multiple directions. A ground breaking technique for bit-level permutation is introduced to improve and swap bit positions in different directions. A hyperchaotic system makes the diffusion matrix. Its starting conditions and parameters come from an SHA-256 hash. In [38] the authors demonstrated the efficiency of Lorenz-based dynamics in achieving strong key dependence and secure pixel-level scrambling. Building upon these foundations, our proposed scheme introduces a dependent bit-level permutation and diffusion mechanism that establishes strong inter-bit coupling and high sensitivity without increasing computational complexity. In this way, the proposed method overcomes the limitations of existing Lorenz-based encryption techniques—such as static diffusion and repetitive multi-round operations while achieving improved security and computational efficiency.

The proposed scheme achieves secure image encryption through the synergistic integration of a 4D Lorenz system with a Radius–Angle Scrambling mechanism and an efficient diffusion process. Unlike many existing methods that rely on multi-round operations to enhance randomness, our approach attains comparable or superior security performance with a single-round structure, thereby reducing computational overhead and improving suitability for real-time applications. It is important to note that the encryption scheme being independent from plaintext characteristics is a fundamental property of all secure cryptographic systems and is not claimed as a unique contribution in this work. Instead, the novelty of our method lies in its ability to combine algorithmic complexity with implementation simplicity, ensuring robust statistical resistance (e.g., NPCR, UACI, entropy, and correlation tests) while maintaining high efficiency. Furthermore, the introduction of the Radius–Angle Scrambling component provides an additional level of permutation diversity that enhances security against conventional cryptanalytic attacks, as demonstrated in our comparative evaluation.

Image encryption plays a crucial role in protecting visual information transmitted across insecure communication channels. Chaotic systems have been widely adopted in image encryption owing to their sensitivity to initial conditions, ergodicity, and deterministic randomness. However, many traditional approaches rely solely on linear or pixel-wise permutations, which may be insufficient to resist advanced cryptanalytic or chosen-plaintext attacks. To overcome these challenges, this study introduces a Circular Radius–Angle Transformation (CRAT) within a four-dimensional (4D) hyperchaotic encryption framework. The central idea of CRAT is to employ random sequences of radius and angle values, generated by the hyperchaotic system, to determine new pixel positions in a circular spatial domain. By transforming pixels from the Cartesian plane into dynamically randomized circular coordinates, the proposed method achieves highly nonlinear pixel redistribution. This enhances diffusion and confusion simultaneously, ensuring improved resistance to statistical, differential, and noise attacks, while maintaining high encryption efficiency.

This article introduces a novel image cryptosystem technique that depends on hyperchaos maps, which integrates simplicity in designing high-complexity algorithms and efficiency by utilizing the characteristics of a chaos system, such as initial conditions sensitivity, parameter sensitivity, and pseudo-number sequence randomicity, among others. Furthermore, the study aims to introduce one assessment approach for scrambling degrees depending on position correlation, which closely matches the subjective evaluation finding and meets a high level of security requirements in terms of efficiency, complexity, reliability, durability, and performance. Given all this, we propose a novel image cryptosystem technique that relies on a hyperchaotic map, specifically the 4D Lorenz system.

The following are the contributions of this work:

(1) Diffusion and confusion levels are achieved using a new technique based on radius, angle, and XOR operation for effective scrambling and replacement procedures.
(2) Bitwise and pixel scrambles are used to increase permutation efficiency.
(3) A hyperchaotic map of Lorenz’s system is employed to generate the pseudo-random keys.

The rest of this study is organized as follows. Section 2 explains the concept of the hyperchaotic system. The proposed algorithm with key generation, confusion, and diffusion stages is described in Section 3. In Section 4, the proposed method is evaluated based on security analyses. The conclusion is presented in section 5.

## 2 Hyperchaotic system

A hyperchaos system has a positive Lyapunov exponent greater than one, indicating that it has extended in multiple directions, resulting in a more complex attractor. The behavior of a whiff of smoke and ocean turbulence are examples of chaotic systems. Chaos systems are notoriously sensitive to their initial circumstances. Rossler originally described hyperchaotic systems in 1979 [39], and the hyperchaotic Rossler system was introduced. Hyperchaotic maps have more intricate dynamics and structures than conventional chaotic maps because the positive Lyapunov exponents have more than two directions in high-phase space. Hyperchaotic systems have a wide range of applications in digital information, as well as secure communication and image encryption. As a result, hyperchaotic systems have become more relevant in nonlinear dynamic research [11]. In this work, we employ a single chaotic generator, namely the 4D Lorenz hyperchaotic system, which is a continuous-time hyperchaotic system characterized by four coupled nonlinear differential equations. The generated chaotic sequences are utilized to drive the scrambling and diffusion processes of the encryption algorithm.

### 2.1 Lorenz system

The Lorenz system is a set of ordinary differential equations for a continuous dynamic system. Hyper-chaos system manners are more complicated than one- and two-dimensional chaos systems. The hyperchaotic system provides multiple positive Lyapunov exponents. As a result, predicting the behavior is more difficult than a chaotic system with lower dimensions. To achieve a hyper-chaos algorithm, there are essential requirements: first, the dimension of the system must not be less than four; second, the equations must have a minimum of two variables, and nonlinear functions must be at least one [40]. The 4D Lorenz system has been chosen over other chaotic maps, such as Chen, Rössler, and hybrid systems, due to its superior hyperchaotic properties and cryptographic suitability. Unlike the Chen or Rössler systems, which typically generate only one positive Lyapunov exponent, the 4D Lorenz system often exhibits two or more positive Lyapunov exponents, signifying hyperchaos and providing a higher degree of unpredictability and sensitivity to initial conditions, essential attributes for encryption applications [41]. In addition to the Lyapunov criterion, the 4D Lorenz formulation presents an extensive hyperchaotic regime, as illustrated by Leutcho et al. [42]; their examination of the multistable 4D hyperchaotic Lorenz system validated its diverse behavioral spectrum encompassing hyperchaotic, chaotic, periodic, and almost periodic states through the modification of a single parameter demonstrating a robustness that maintains the system in a hyperchaotic state without necessitating meticulous fine-tuning. The Chen and Rössler variations do not regularly attain this parametric stability. The 4D fractional-order Chen hyperchaotic system has been effectively utilised for colour image encryption [43]; however, its implementation necessitates the integration of various cryptographic elements such as sine maps, S-boxes, and hybrid DNA coding to address its limited dynamic range, resulting in heightened complexity without a corresponding enhancement in security. Moreover, running hyperchaotic systems at fractional order allows for an increase in the number of control variables and an expanded key space; nevertheless, this advantage incurs a substantial computing burden that is unwarranted for conventional image encryption applications [10]. The integer-order 4D Lorenz hyperchaotic system achieves a significant equilibrium between security and efficiency, fulfilling the requirements of a resilient encryption scheme, which encompasses an extensive key space and resistance to statistical, differential, and chosen/known-plaintext attacks, all while preserving low computational complexity that renders it appropriate for practical implementation [44]. The expansion to four dimensions significantly enlarges the key space, with recent implementations achieving magnitudes exceeding 10^150^, far beyond the 2^100^ threshold generally required for resisting brute-force attacks [45]. In contrast, Chen and Rössler systems, although simpler, are limited in their attractor structures and cannot offer such extensive key spaces, which makes them comparatively weaker in cryptographic resistance. Hybrid systems, while capable of combining features of multiple chaotic models, often introduce additional mathematical and computational complexity, resulting in higher resource requirements and reduced efficiency for real-time encryption. Concerning higher-dimensional alternatives (5D and above), although they provide supplementary positive Lyapunov exponents, the increased complexity in parameter calibration and hardware execution surpasses the minimal security enhancement for standard image encryption applications. The aforementioned factors—a superior Lyapunov exponent count compared to 3D systems, an extensive hyperchaotic parameter range, a competitive key space, and a moderate computational cost—substantiate the selection of the 4D Lorenz hyperchaotic system as the optimal choice in this study, aligning with the methodologies employed in the cited research [10,42–44].

**Fig 1:**
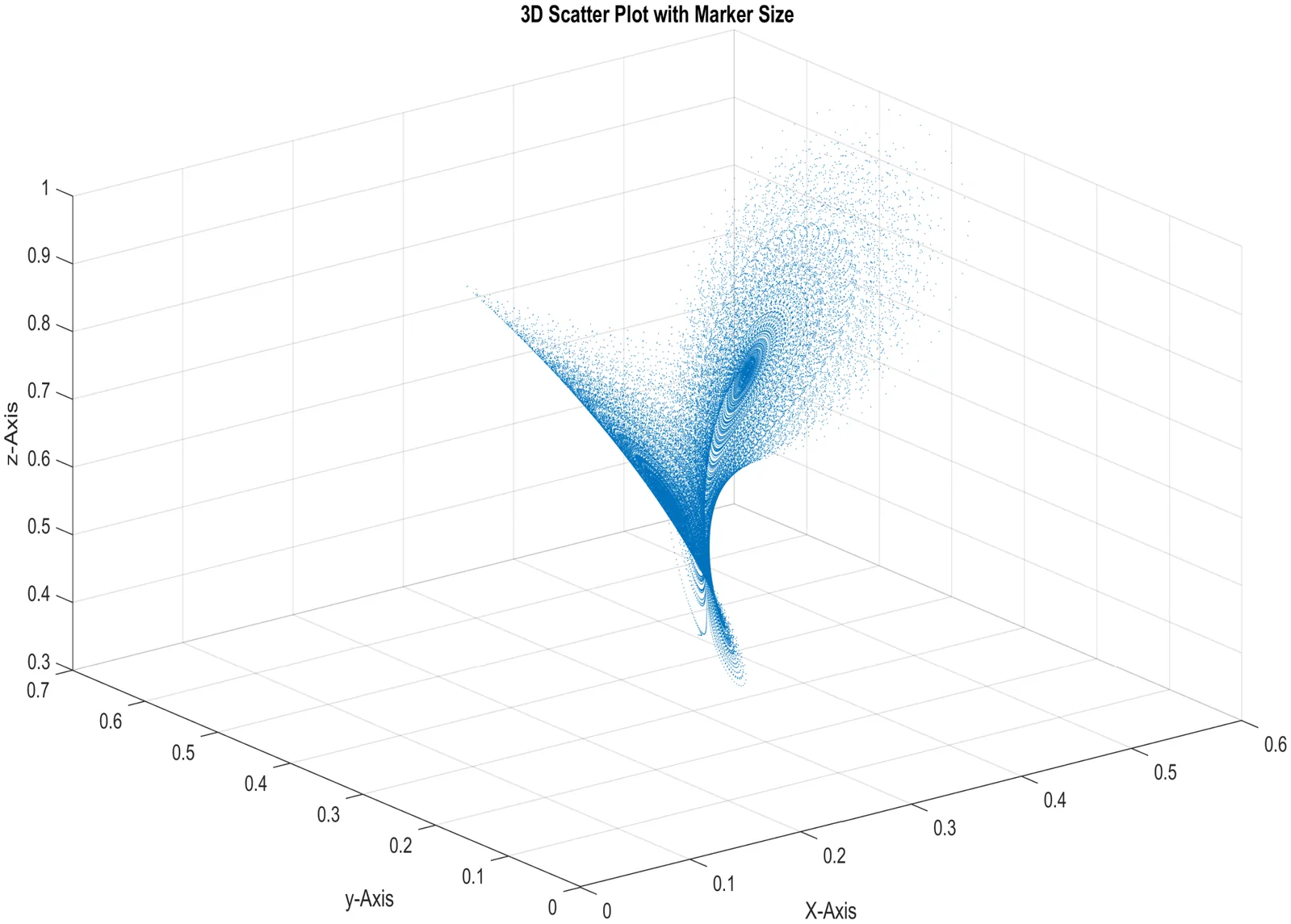
Lorenz system simulation results from their projections in x-y-z plane.

**Fig 2:**
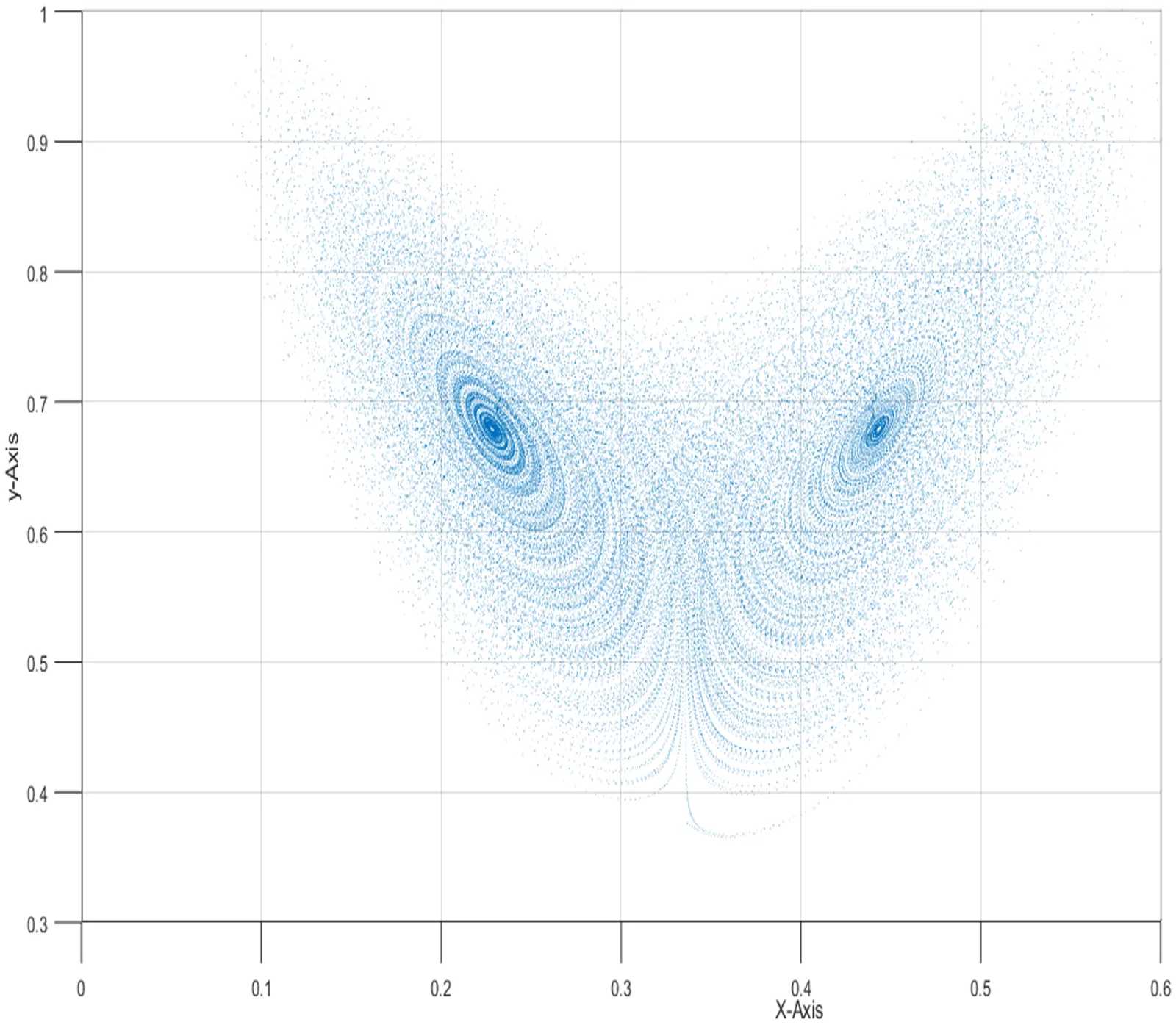
Lorenz system simulation results from their projections in x-y.


In a chaotic system, small changes in initial conditions can lead to significantly different outcomes over time, making long-term prediction and control complex. Chaotic systems are deterministic, which means their initial conditions and parameters entirely determine their hyperchaotic behaviour [40], as created by the Lorenz system. Appending a new nonlinear controller W to the three-dimensional Lorenz system identified by equation (1) will achieve a new four-dimensional hyperchaotic map [46–48]. Figs 1 and 2 illustrates the drawing of the 4-dimensional hyperchaotic Lorenz system attractor.

$$\begin{array}{c} \text{4D Lorenz system}\left\{ \begin{array}{c} @l\frac{dx}{dt}=\sigma (y-x),\\ \frac{dy}{dt}=x(\rho -z\;)-y+w,\mathrm{\backslash vspace}1mm\\ \frac{dz}{dt}=xy-\beta z\;,\mathrm{\backslash vspace}1mm\\ \frac{dw}{dt}=\;-\gamma x. \end{array}\right. \end{array}$$ (1)

**Fig 3:**
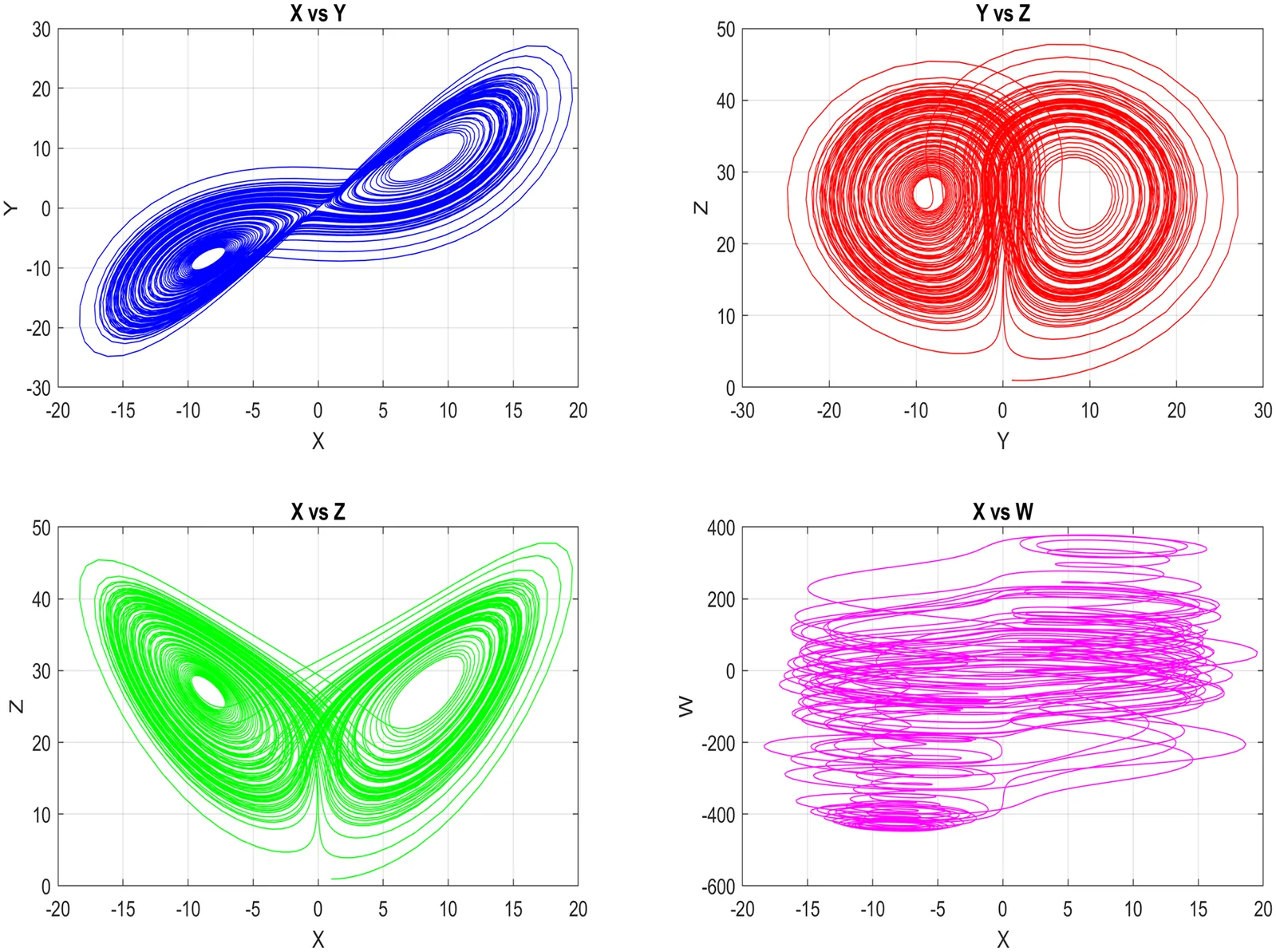
Attractor trajectory for 4-D Lorenz system.

**Fig 4:**
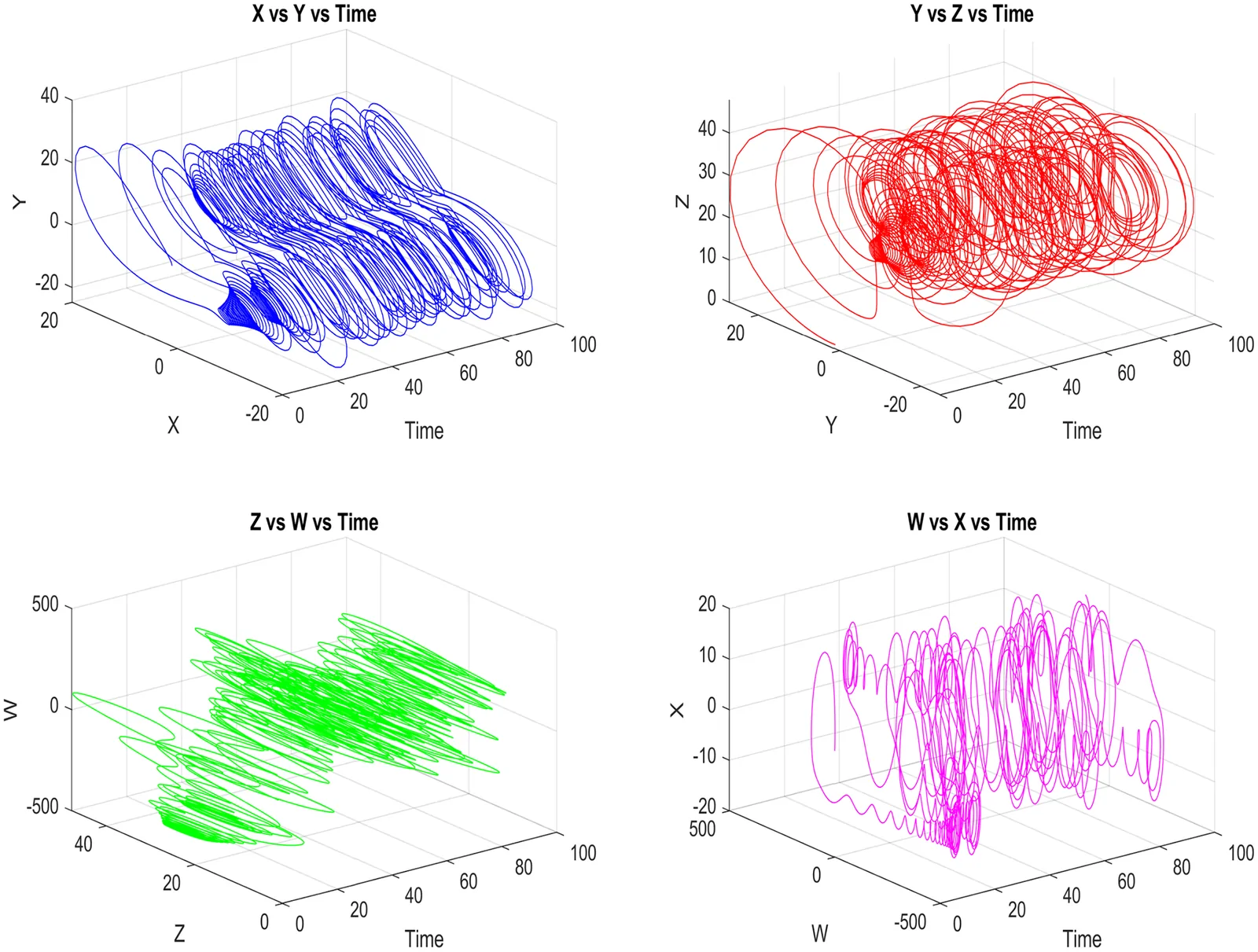
Phase diagram for 4-D Lorenz system.

Here, x, y, z, and w are initial conditions, and the control parameters are σ, β, γ, and ρ. Fig 3 shows the attractor trajectory and Fig 4 shows phase diagram for the four-dimensional chaotic Lorenz system.

### 2.2 Lyapunov exponent (LE)

**Fig 5:**
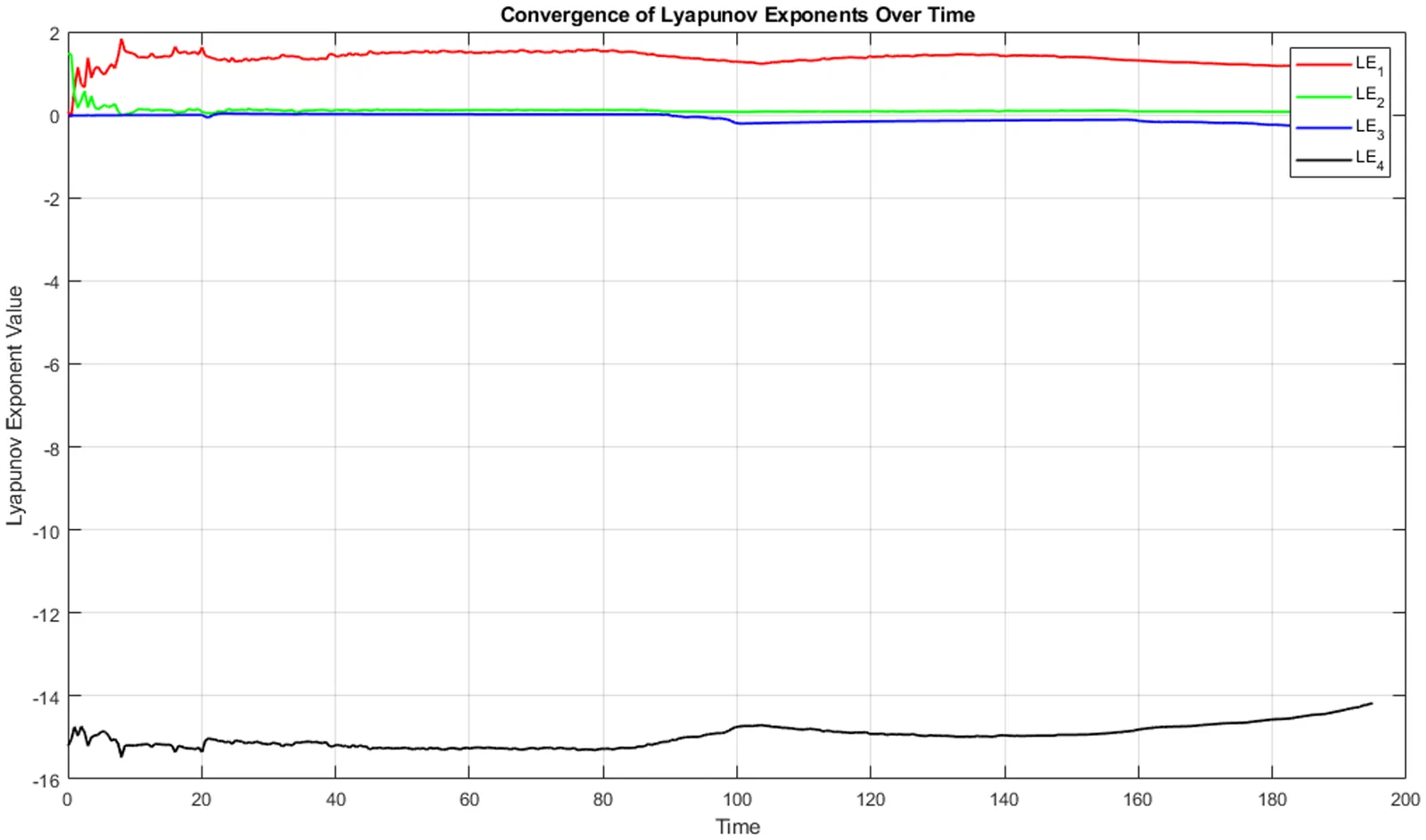
Convergence of the four Lyapunov exponents (LEs) of the proposed method.

LE is a fundamental metric that indicates the level of chaos inside a dynamic system. It delineates the mean velocity at which trajectories in the system’s phase space, which are in proximity, diverge. A positive Lyapunov exponent indicates that the system exhibits high sensitivity to initial conditions, which means chaotic behaviour. A negative or zero value indicates that the dynamics are periodic or nearly periodic [49]. In chaotic encryption systems, the Lyapunov Exponent (LE) is a crucial metric for assessing the randomness and complexity of the generated sequences. It confirms that even slight alterations to the initial settings result in significantly varied outcomes; therefore, systems exhibiting multiple positive Lyapunov exponents are classified as hyperchaotic, offering higher entropy and enhanced diffusion properties suitable for robust cryptographic applications [50]. Fig 5 illustrates the convergence of the four Lyapunov exponents (LEs) of the proposed four-dimensional Lorenz-type chaotic system over time. As shown, the trajectories of the Lyapunov exponents stabilize after an initial transient phase, confirming numerical convergence and the existence of a stable attractor. The final computed Lyapunov spectrum is [1.0659, 0.1133, −0.7937, −13.7064]. The presence of two positive Lyapunov exponents indicates that the system exhibits hyperchaotic behaviour, characterized by exponential divergence along two independent directions in phase space. The third exponent is negative but close to zero, suggesting weak contraction, while the large negative fourth exponent reflects strong dissipative dynamics. Overall, the figure demonstrates that the proposed system is both hyperchaotic and dissipative, possessing complex dynamics with sensitive dependence on initial conditions, which is an essential property for secure and unpredictable cryptographic key generation.

### 2.3 Contribution of the Radius–Angle Scrambling Component

To rigorously quantify the contribution of the Radius–Angle Scrambling component, a controlled ablation study was conducted. Two system variants were evaluated: V1, the complete proposed method; and V2, an otherwise byte-identical system from which only the Radius–Angle Scrambling stage is removed, routing the plaintext image directly to the XOR diffusion stage. The Lorenz parameters, initial conditions, key derivation, and diffusion operations are identical in both variants, ensuring that any measured performance difference is attributable solely to the scrambling component. Results are summarised in Table 1. Diffusion-governed metrics are effectively equivalent across both variants, as expected. Mean entropy is 7.9991 (V1) and 7.9990 (V2), a difference of 0.000132 bits that lies within measurement precision. NPCR values of 99.61% and 99.62% both exceed the accepted security threshold of 99.6094%, and the UACI of V1 (33.4571%) is marginally closer to the theoretical ideal of 33.4635% than that of V2 (33.4741%). Bit plane complexity is 0.4997 versus 0.4996, both within 0.0004 of the ideal 0.5000. These results confirm that the Lorenz diffusion engine operates consistently regardless of upstream scrambling and that both variants satisfy conventional cryptographic benchmarks for diffusion-based security. The decisive divergence emerges across all positional security metrics. In V2, with no scrambling applied, the scrambling degree is 0.00%, the mean pixel displacement is 0 px, Pixel Position Tracking Resistance (PPTR) is 0.000, and the post-scrambling neighbour distance is 1 px—identical to the original pre-encryption pixel separation. XOR diffusion alone preserves the complete spatial layout of the plaintext image in the cipher: every encrypted pixel occupies its original spatial position, and all neighbourhood relationships remain intact. An adversary in possession of the cipher image can therefore determine the spatial origin of any encrypted value without key material, rendering the scheme vulnerable to position-based structural analysis. The Radius–Angle Scrambling component eliminates this vulnerability categorically. In V1, 99.39% of pixels are displaced from their original positions, with a mean displacement of 567.56 px and a maximum of 1,597.98 px, corresponding to long-range scattering produced by the polar coordinate transformation x’ = r·cos(θ), y’ = r·sin(θ), which couples pixel positions by radial distance rather than Cartesian proximity. The PPTR rises to 0.857, with 89.77% of pixels displaced beyond 25% of the image diagonal. Most critically, the post-scrambling neighbour distance increases from 1 px to 414.98 px—a 414-fold increase—demonstrating that originally adjacent pixels are scattered across nearly one-third of the image diagonal on average, completely destroying the local spatial structure of natural images and precluding any neighbourhood-based reconstruction from the cipher. The PPTR standard deviation of 0.5057 further indicates that displacement is highly irregular and heterogeneous, a consequence of the transcendental cosine and sine functions used in key derivation, which produce analytically non-invertible distributions without knowledge of the exact Lorenz trajectory. Additionally, V1 achieves a faster encryption time (1.09 sec) than V2 (1.86 sec), confirming that the Radius–Angle Scrambling stage introduces no computational overhead and yields a net performance advantage owing to the efficiency of the vectorised sort-based polar shuffle. The elimination of the Radius–Angle Scrambling component results in a complete failure of all positional security measurements, whereas diffusion-based metrics remain unaltered. Its existence results in extensive, irregular, long-range pixel displacement that is intentionally non-invertible analytically. The results affirm that the Radius–Angle Scrambling component is a crucial and distinctly effective aspect of the proposed encryption technique, whose contribution cannot be duplicated by XOR diffusion alone.

**Table 1:** V1 (with radius-angle scrambling) vs V2 (without radius-angle scrambling).

Metric	V1 (Proposed)	V2 (No Scrambling)	Verdict
**ENTROPY & DIFFERENTIAL ATTACK**			
Mean entropy (ideal = 8.000)	**7.9991**	7.9990	*V1 superior*
NPCR (%)	99.6139	99.6225	*≈ Equal*
UACI (%)	**33.4571** ✓	33.4741	*V1 superior*
**PIXEL CORRELATION (|COEFF|, IDEAL = 0.000)**			
Horizontal	**0.0031**	0.0208	*V1 superior*
Vertical	**− 0.0036**	0.0113	*V1 superior*
Diagonal	**0.0019**	0.0025	*V1 superior*
**SCRAMBLING & POSITIONAL SECURITY**			
Scrambling degree (%)	**99.3941**	0.0000	*V1 decisive*
Mean displacement (px)	**567.56**	0.00	*V1 decisive*
PPTR mean (0–1)	**0.8566**	0.0000	*V1 decisive*
PPTR rate >25% diag (%)	**89.77**	0.00	*V1 decisive*
Neighbour dist. after (px)	**414.98**	1.00	*V1 decisive*
**BIT PLANE COMPLEXITY & SPEED**			
Mean BP complexity (ideal = 0.500)	0.4997	0.4996	*≈ Equal*
Encryption time (sec)	**1.0948**	1.8638	*V1 superior*

## 3 Proposed scheme

**Fig 6:**
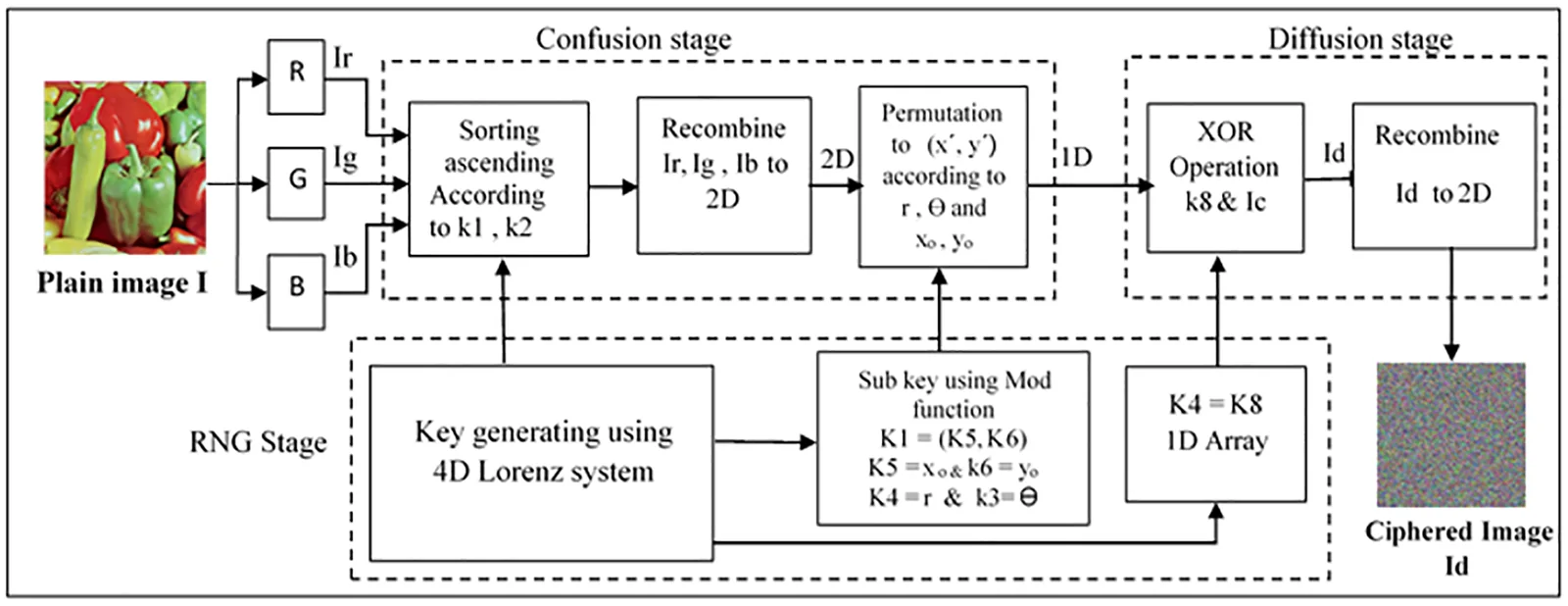
Block diagram of the encryption process for the proposed method.

**Fig 7:**
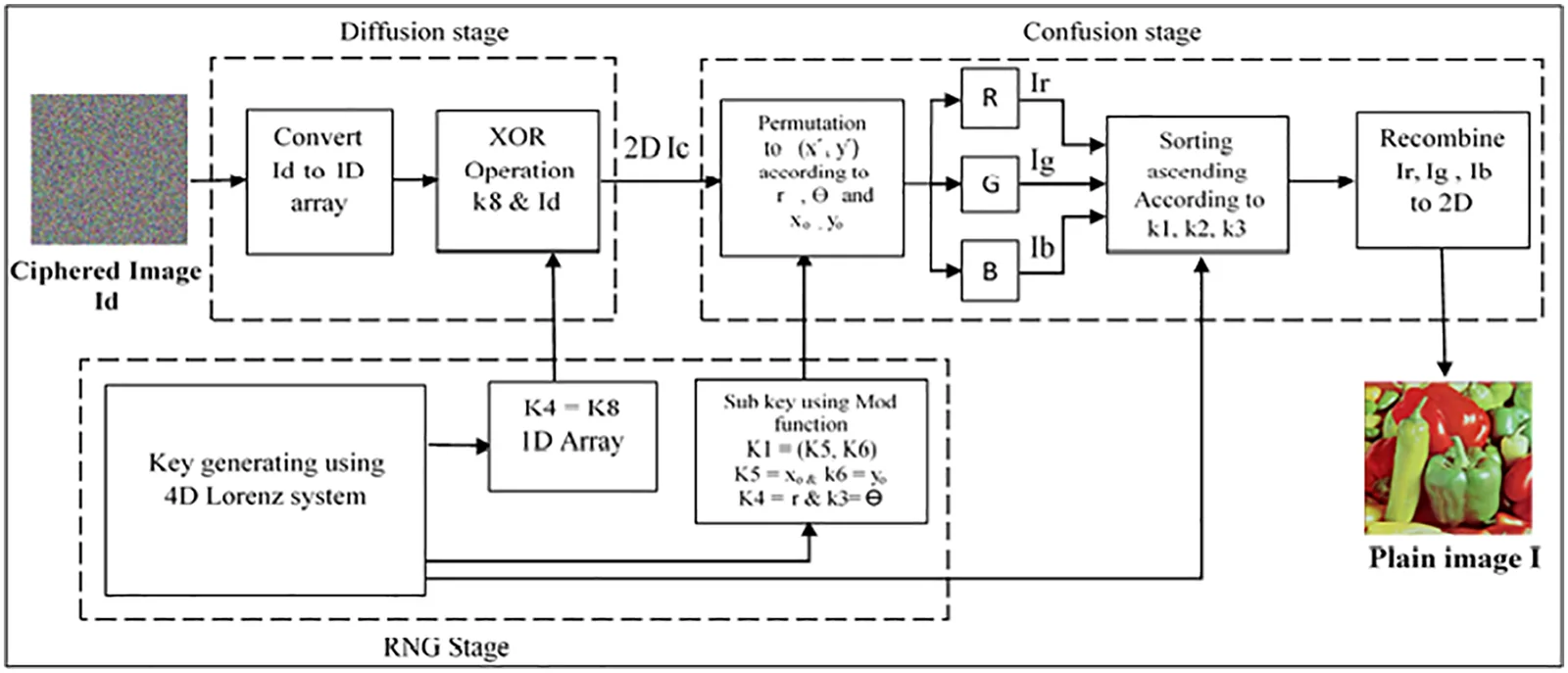
Block diagram of the decryption process for the proposed method.

Fig 6 illustrates the framework for encryption in the proposed cryptosystem method, and Fig 7 represents the decryption process. The main processes utilized in the proposed method are image preprocessing, keys matrix generation, confusion, and diffusion processes. The following stages and subsections are explained in detail.

### 3.1 Pre-processing

This section presents the process for selecting and decomposing the image before any further processing.

1. Selection of image: First, an input image I with a dimension of (m * n) is taken from the specified dataset USC-SIPI.
2. Image type check:
  - If I (m * n) is a grayscale image, then we will use it immediately

$$\begin{array}{c} {I\;}_{\text{8}-bit}=I_{\;gray} \end{array}$$

- If I (m * n) a color image has 24-bit Red, Green, Blue it will be separated to its individual channels, Ir, Ig and Ib.

$$\begin{array}{c} I_{RGB}=\;\left\{I_{r},I_{g}.I_{b}\right\} \end{array}$$

Each channel, Ir, Ig, and Ib, is saved in an 8-bit array with the same dimensions as the original image I (m * n).


$$\begin{array}{c} {\;I}_{r,}I_{g},\;I_{b}\;\in R_{\text{8}-bit}^{m\;x\;n} \end{array}$$

### 3.2 Keystream generator

Keystream generation is critical in image encryption systems, and hyper-chaos maps are employed in the suggested method. For the diffusion and confusion stage, the dynamic of the Lorenz system was utilized to produce four pseudo-random sequences of keys, which will be created by adding a new linear variable to the Lorenz map. The seeds of the 4D Lorenz system and its control parameters are generated using the SHA-256 hash of the plaintext image combined with the secret key. The hash digest is segmented, normalised, and allocated to the state variables and parameter ranges σ∈(8,28), ρ∈(20,40), β∈(2,5), and α∈(0.5,5). This guarantees that the chaotic system functions only inside the chaotic regime while preserving heightened sensitivity to both the key and plaintext. The range of key values will be between 0 and 1; the analysis tests indicated that the Lorenz system exhibited hyperchaos and stability across a broad range of parameters. In the Lorenz attractor, the geometric structure demonstrated that the double-lobe structure persists in the system’s 4D space of attractor [36]. The suggested dynamical system is represented by the following linearly expanded Lorenz equation (2).


$$\begin{array}{c} \begin{array}{c} dx=\sigma \;\left(\;y_{i-\text{1}}-x_{i-\text{1}}\right)*dt\;,\\ dy={(\;x}_{i-\text{1}\;\cdot }\;\left(\;\rho -\;z_{i-\text{1}\;}\right)-\;y_{i-\text{1}\;})*dt\;,\\ dz=\left(\;x_{i-\text{1}\;}*\;y_{i-\text{1}\;}-\;\beta *\;z_{i-\text{1}\;}\right)*dt,\\ dw=\left(-\;\gamma -\;x_{i-\text{1}\;}\right)*dt. \end{array} \end{array}$$ (2)

Where σ is a constant that affects the rate of change of x. ρ, β, and γ are other constants affecting the rates of change of y, z, and w, respectively, and dt is the time step.

The *x(i), y(i), z(i*), and *w(i)* represent the variables values at the current time, and *x(i − 1), y(i − 1), z(i − 1), and w(i − 1*) are the values at the earlier time step *i − 1.*

After calculating the rates of change *dx, dy, dz,* and *dw*, the values are updated in equations 3, 4.


$$\begin{array}{c} \begin{array}{c} x_{i}=\;x_{i-\text{1}\;}+dx,\\ y_{i}=\;y_{i-\text{1}}+dy,\\ z_{i\;}=z_{i-\text{1}}+dz,\\ w_{i}=\;\gamma +dw, \end{array} \end{array}$$ (3)

$$\begin{array}{c} \begin{array}{c} x_{i}=x_{i-\text{1}\;}+\;\sigma \;\left(\;y_{i-\text{1}}-x_{i-\text{1}}\right)*dt\;\\ y_{i}=y_{i-\text{1}\;}+\;{(\;x}_{i-\text{1}\;\cdot }\;\left(\;\rho -\;z_{i-\text{1}\;}\right)-\;y_{i-\text{1}\;})*dt\;,\\ z_{i}=\;z_{i-\text{1}\;}+\;\left(\;x_{i-\text{1}\;}*\;y_{i-\text{1}\;}-\;\beta *\;z_{i-\text{1}\;}\right)*dt\;,\\ w_{i}=\;\gamma \;-\;\gamma -\;x_{i-\text{1}\;}*dt\;, \end{array} \end{array}$$ (4)

Each variable x, y, z, and w is then normalized using the formula (5):


$$\begin{array}{c} \text{Normalized }-\text{ value }=\frac{Value\;-\text{ min}\;Value}{\text{max}Value\;-\;\text{min}Value} \end{array}$$ (5)

#### 3.2.1 Random keys generating process.

Primary pseudo-random number sequences are generated using the 4D Lorenz system, represented as k = {k1, k2, k3, k4}. Specifically, use k1, k2, and k3 with the color channels red, green, and blue (Ir, Ig, and Ib) in succession. From k1, the subkeys k5 and k6 are derived by dividing k1 by scaling. Indices are calculated using equation 6 to derive k5 and k6, employing the mod function as described in equation 7.


$$\begin{array}{c} k_{\text{5}\;}=\;\left(\frac{k_{\text{1}\;}}{scaling\;indices}\right), \end{array}$$ (6)

$$\begin{array}{c} {\;k}_{\text{6}\;}=mod\;\left(k_{\text{1}}\;,\;scaling\;indices\;\right), \end{array}$$ (7)

The scaling of indices is based on the sizes of the images.

#### 3.2.2 key distribution.

To ensure secure synchronization of the chaotic key generation process, quantum key distribution (QKD) protocols like BB84 [44] are utilized to transmit the initial conditions and control parameters of the 4D Lorenz system between the communicating parties. Instead of directly distributing the chaotic key stream, which may expose it to interception, the BB84 protocol enables the establishment of a quantum-secure channel through which only the sensitive parameters are exchanged. Once these parameters are securely shared, both parties can independently regenerate identical chaotic sequences locally using the 4D Lorenz system, thereby eliminating the need to transmit the entire key. This hybrid approach was adopted because it combines the large key space and hyperchaotic properties of the 4D Lorenz system [44] with the provable information-theoretic security of QKD. By transmitting only the parameters via BB84 and regenerating the chaotic key locally, the scheme ensures minimal information leakage, reduced communication overhead, and enhanced resilience against both classical and quantum adversaries.

## 4 Confusion phase

In the image encryption framework, the confusion stage is responsible for resolving the correlation. In an original image, the correlation is very high among the close pixels because of the closeness of pixel values [37]. The manner to resolve the correlation is by performing the modification of pixel positions in the original image, utilizing the created sequence of random keys to maintain control over the entire process at this phase.

- Sorting of a random sequences k_1_, k_2_, k_3_ in ascending or descending order.

$$\begin{array}{c} k_{\text{1}}^{sorted}\;,\;k_{\text{2}}^{sorted}\;,\;k_{\text{3}}^{sorted} \end{array}$$

- Permute the pixel locations in each channel R, G, B, based on sorted indices order of $k_{\text{1}}^{sorted}\;,\;k_{\text{2}}^{sorted}\;,\;k_{\text{3}}^{sorted}$, using equation 8.

$$\begin{array}{c} \begin{array}{c} I_{r}^{scrambled}=permute\;(\;Ir\;,\;k_{\text{1}}^{sorted}\;),\\ I_{g}^{scrambled}=permute\;\left(\;Ig\;,\;k_{\text{2}}^{sorted}\;\right),\\ I_{b}^{scrambled}=permute\;\left(\;Ib\;,\;k_{\text{3}}^{sorted}\;\right), \end{array} \end{array}$$ (8)

**Fig 8:**
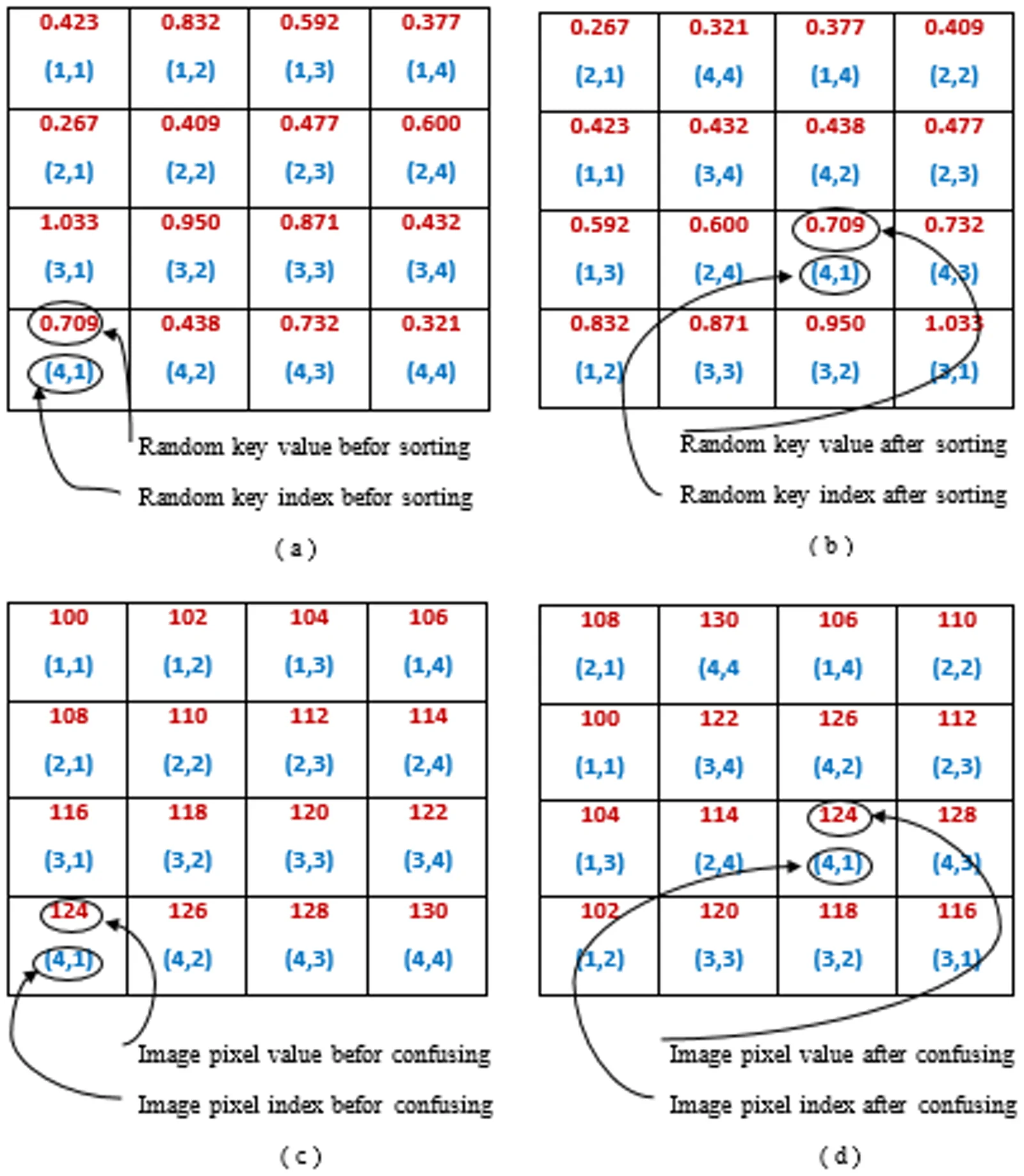
Confusion phase pixel’s position after first scrambling.


- The first scrambled image channels ${\;I}_{r}^{scrambled\;},\;I_{g}^{scrambled}\;,\;I_{b}^{secrambled}$ are the output of first confusion process. Fig 8 illustrates the pixel’s position after first scrambling.

$$\begin{array}{c} I_{scrambled}=\;\left\{I_{r}^{scrambled}\;,\;I_{g}^{scrambled}\;,\;I_{b}^{scrambled}\right\} \end{array}$$

- k4 and k5 represent the initial coordinates (x^0^, y^0^), and k6 represents the radius r, which depends on a dimension of the image I (m * n), and k7 represents the angle θ, which can range 0 to 2π.

$$\begin{array}{c} \begin{array}{c} x^{\text{0}}=k_{\text{4}}\;,y^{\text{0}}=k_{\text{5}}\\ r=k_{\text{6}}\text{ where }r\leq \;\sqrt{M^{\text{2}\;}+\;N^{\text{2}}}\;\\ \theta =k_{\text{7}}=k_{\text{3}}\text{ where }\text{0}\;\leq \;\theta \;<\;\text{2}\pi \; \end{array} \end{array}$$

- The new coordinates $\;\left(x^{\prime }\;,\;y^{\prime }\right)$ of pixels are calculated using equations 9 and 10 based on the radius r and angle θ from a pseudo-random key generated, and the initial coordinates $\left(x^{\text{0}},\;y^{\text{0}}\right)$ that are selected randomly based on keys are as follows:

$$\begin{array}{c} x^{\prime }=\;x^{\text{0}}+r\;\text{cos}\theta , \end{array}$$ (9)

$$\begin{array}{c} y^{\prime }=\;y^{\text{0}}+r\;\text{sin}\theta ,\; \end{array}$$ (10)

- The ciphered image (Ic) obtained after applying this transformation will have pixels at new coordinates (x′, y′), which leads to the uncorrelation between the adjacent pixels, which considers the second scrambling.

## 5 Diffusion phase

The diffusion phase includes three main factors: the fourth dynamic key (k8), the Ic, and the XOR operation. The major reason for the diffusion stage is to eliminate the Ic statistical data by modifying the values of image pixels with a convenient technique.

- The k8 key is generated from a fractal number of K from the earlier section, the range between 0 and 1, with accuracy 10^15^, which are then converted to integer values in the range 0 to 255 and then converted to binary form.

$$\begin{array}{c} {\text{k}}_{\text{4}}={\text{k}}_{\text{8}}=[\text{0}-\text{1}]\;\Rightarrow \text{ k}\text{8}=\;[\text{0}-\text{2}\text{5}\text{5}] \end{array}$$

- The main operation in the diffusion process is the XOR between the scrambled image Ic output from the confusion stage and the generated key k8; the operation is represented by formula (11).

$$\begin{array}{c} I_{d}=\;I_{c}\;⊕\;k_{\text{8}}, \end{array}$$ (11)

After the diffusion stage, the histogram of Id is semi-uniform, meaning that pixel intensities are evenly distributed across possible values.

## 6 Decryption phase

The reverse action of image encryption is termed the decryption process. The following are the procedures to decrypt the ciphered image:

Step 1: Select a ciphered colour image Id with a dimension equal to the original I (m * n).
Step 2: Generate a pseudo-random number sequence of keys using a hyperchaotic map’s Lorenz system, using the same values for initial and control parameters in encryption processing.
Step 3: Diffusion decryption: Perform an XOR operation between the key (k8) and the encrypted image (Id). The resulting Id will correspond to the output from the confusion process (Ic).
Step 4: Confusion Decryption: Reverse the confusion process using the confusion keys k4, k5, and k6. These keys are generated using a hyperchaotic (Lorenz) system and sub key from mod function to reorder$\;\left(x^{\prime }\;,\;y^{\prime }\right)$ to the original position$\left(x^{\text{0}},\;y^{\text{0}}\right)$ in the second scrambling stage.
Step 5: Image Restoration: Use the keys k1, k2, and k3 to resort to the decrypted image Ic for each channel independently, Ir, Ig, and Ib, as the process in the first scrambling stage and restore all channels to their original form.

## 7 Security analysis and numerical results

Ablation research was performed to evaluate the distinct contribution of the CATR component, comparing the proposed encryption method against a baseline variation that uses conventional bit-level permutation without the radius–angle transformation. According to the experimental results, adding the CRAT greatly enhances important security metrics: entropy approaches a maximum value of 8, the correlation between neighbouring pixels is dramatically decreased, and the NPCR and UACI values are closer to theoretical ideals. The findings validate that radius-angle scrambling improves inter-bit diffusion and plaintext sensitivity, therefore augmenting resistance to statistical and differential assaults. The investigation confirms that this component is essential for the superior security performance of the proposed encryption system.

Traditional statistical measurements serve as significant indications of encryption quality; nevertheless, they fail to comprehensively reflect the actual benefits of a technique. The suggested technique attains a compromise between complexity and efficiency by integrating a resilient bit-level, plaintext-dependent permutation with CRAT scrambling, therefore ensuring high diffusion and sensitivity to fluctuations in plaintext. In contrast to multi-round or computationally demanding techniques, the single-pass architecture reduces processing duration, facilitating real-time applications without sacrificing security. The suggested technique sustains competitive security performance while providing practical efficiency, rendering it suitable for applications in real-time image and video encryption, embedded systems, and IoT contexts.

This section evaluates the encrypted image quality by comparing the proposed technique to other techniques discussed in literature. The proposed method is applied using MATLAB R2023a on a computer with 8 GB of RAM and an 8–core Intel® Core TM i3–5010U CPU with a frequency of 2.1 GHz. The datasets were derived from the following public domain resources USC-SIPI employed for processing and comparison.

### 7.1 Key space analysis

Key space indicates the number of tries to guess the right decipher key. Robust encryption must have a ciphering key space not less than 10^30^ ≈ 2^100^,the bits number in a key is represented based on the exponent. To resist brute force attacks, a big key space is required to achieve high-security encryption systems [51]. The 4D chaotic map has a precision of the key is 10^15^, and the keys are x, y, z, w, ρ, β, λ, and σ. Thus, the total possibility of key space is 10^120^ ≈ 2^400^. The key space of the proposed scheme offers high capability against the brute force attack.

### 7.2 NIST randomness experiment

The NIST has released a test suite for the randomness testing of random number and pseudorandom number generators. This test method can be used to analyze the uncertainty of a chaotic sequence in the encryption system and the randomness between the encrypted image pixels. The parameters of NIST SP 800−22 tests in Table 2 illustrate the P-value for all the tests being above 0.01, which indicates that the chaotic sequence has adequate randomness.

**Table 2:** NIST SP 800−22 tests result for the proposed method.

Test name	Average P-Value	Result
Monobit Frequency	0.30094	PASS
Block Frequency	0.67605	PASS
Runs Test	0.15299	PASS
Longest Run of Ones	0.33759	PASS
Binary Matrix Rank	0.71121	PASS
Spectral (FFT)	0.57666	PASS
Non-overlapping Template	0.15783	PASS
Overlapping Template	0.68785	PASS
Maurer’s Universal	0.20173	PASS
Linear Complexity	0.79428	PASS
Serial Test	0.51402	PASS
Approximate Entropy	0.51616	PASS
Cumulative Sums	0.27681	PASS
Random Excursions	0.46731	PASS
Random Excursions Variant:	0.44582	PASS

### 7.3 Key sensitivity analysis

**Fig 9:**
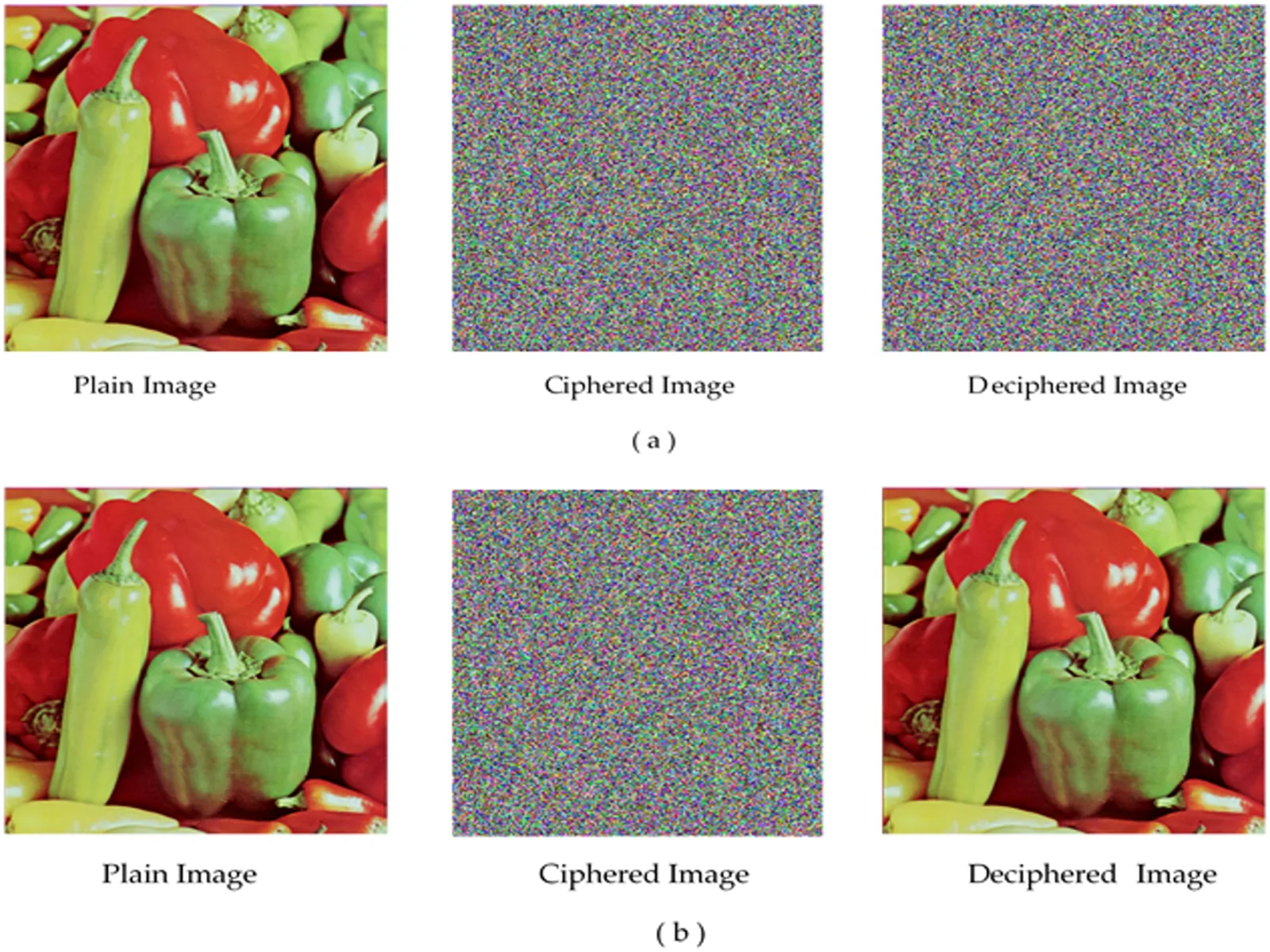
The outcome for the correct, and the incorrect key.

The highly sensitive secret key in cryptography provides robust encryption, and even a small alteration in the secret key results in an incorrectly decrypted image. The robustness of an encryption framework in secure image cryptosystems relies on an evaluation of secret keys. Generally, in a cryptography system, to decrypt an image, the correct key used for encryption must be offered [44]. Chaotic maps are extremely sensitive to parameters and initial conditions, where small changes in them lead to large differences. Fig 9 illustrates the outcome when using correct and incorrect keys with a small change in initial conditions or parameters during decryption. Based on the outcomes, we note that the suggested method has achieved the required goals.

### 7.4 Correlation between Adjacent Pixels

**Fig 10:**
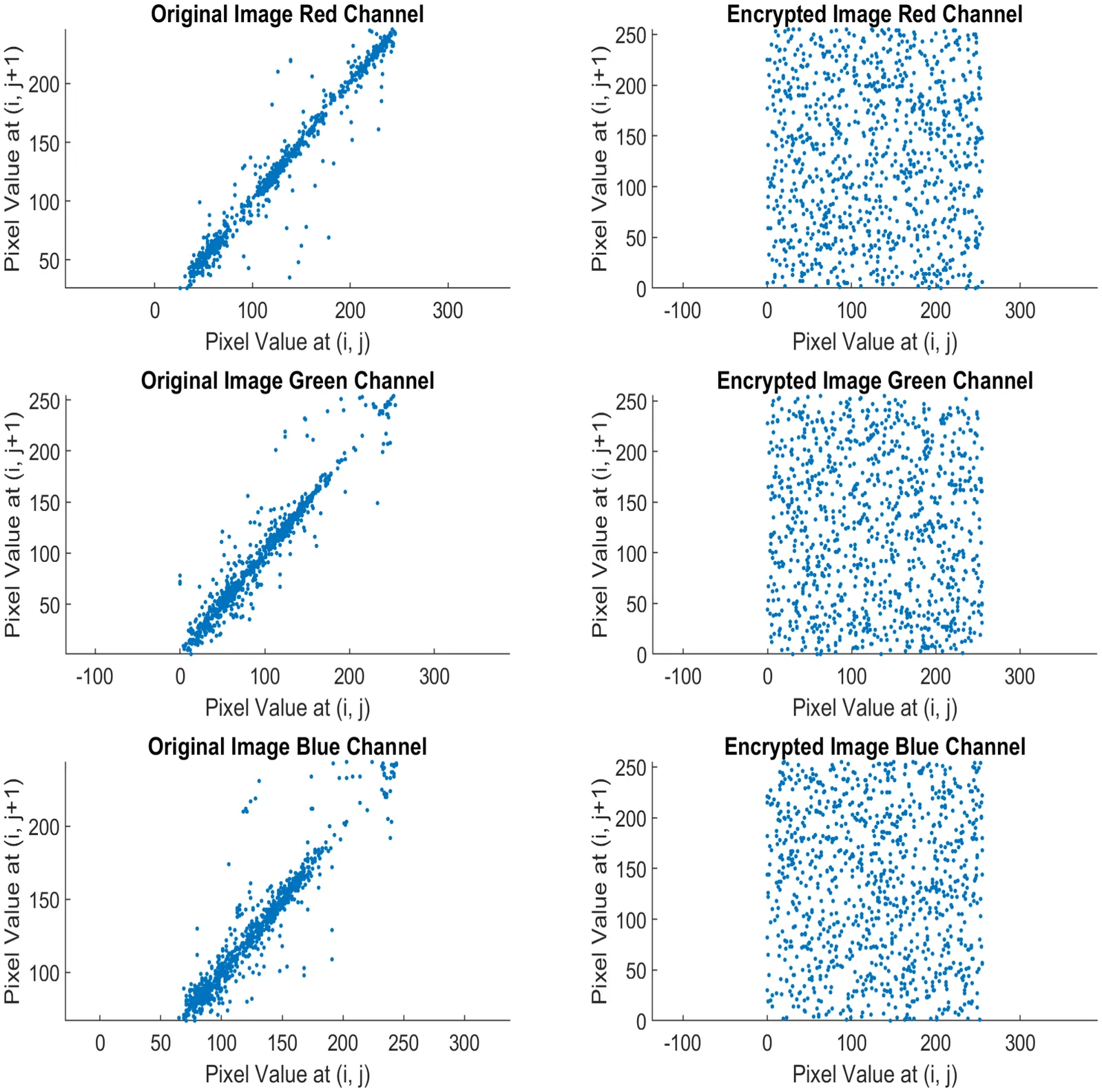
The pixels correlation coefficient of original and encrypted image for Female image.

**Fig 11:**
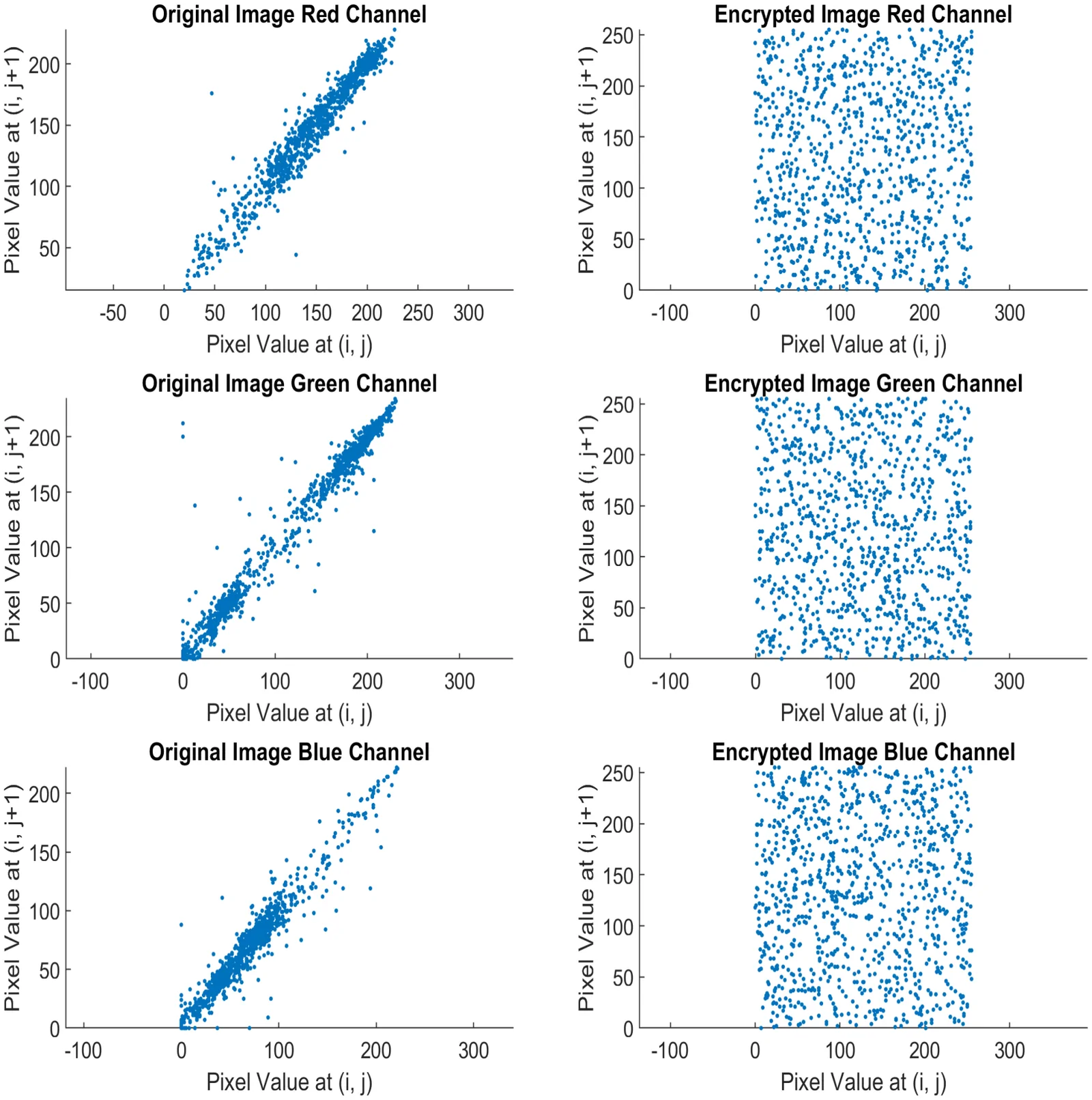
The pixels correlation coefficient of original and encrypted image for Mandrill image.

**Fig 12:**
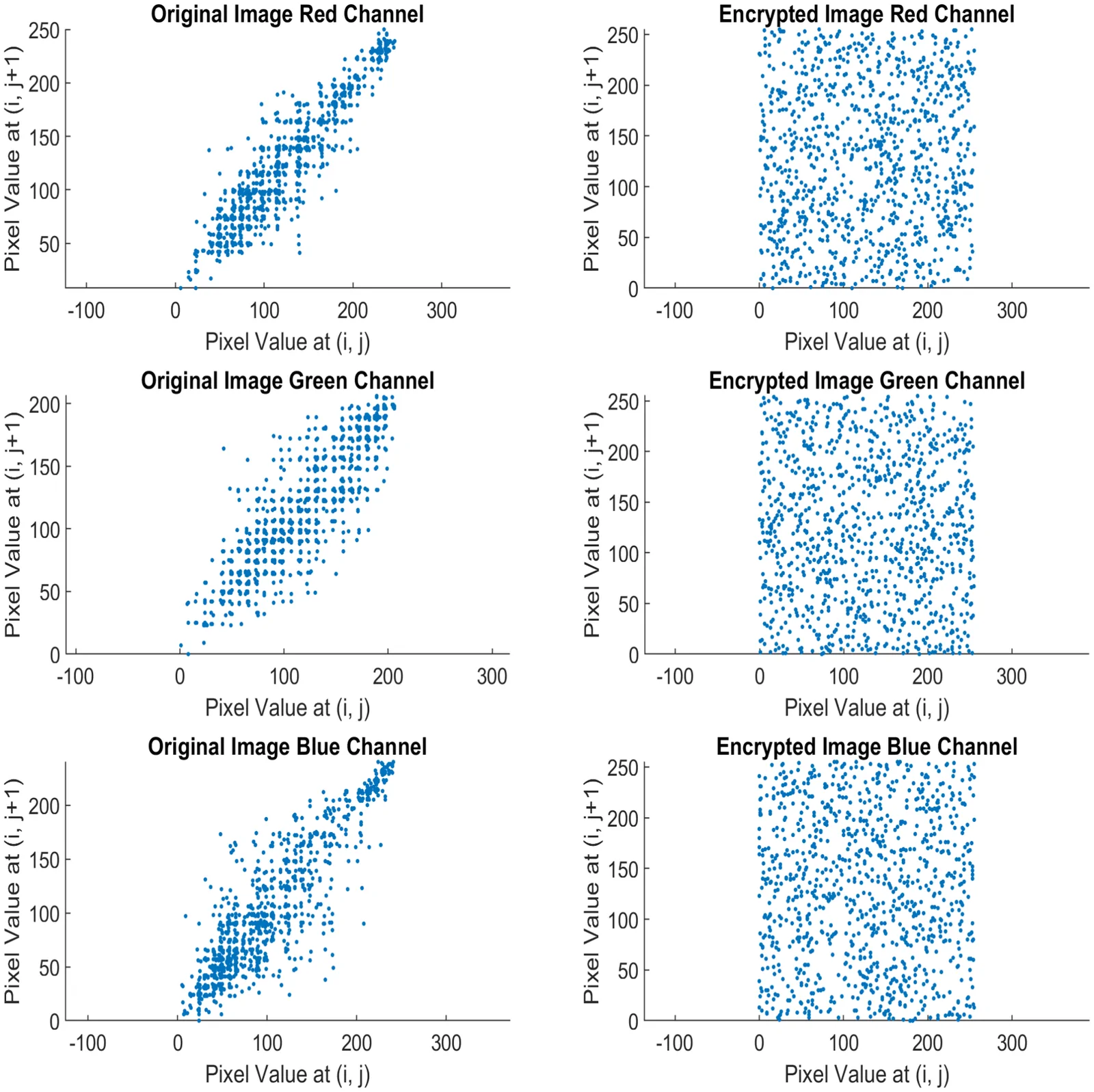
The pixels correlation coefficient of original and encrypted image for Peppers image.

**Fig 13:**
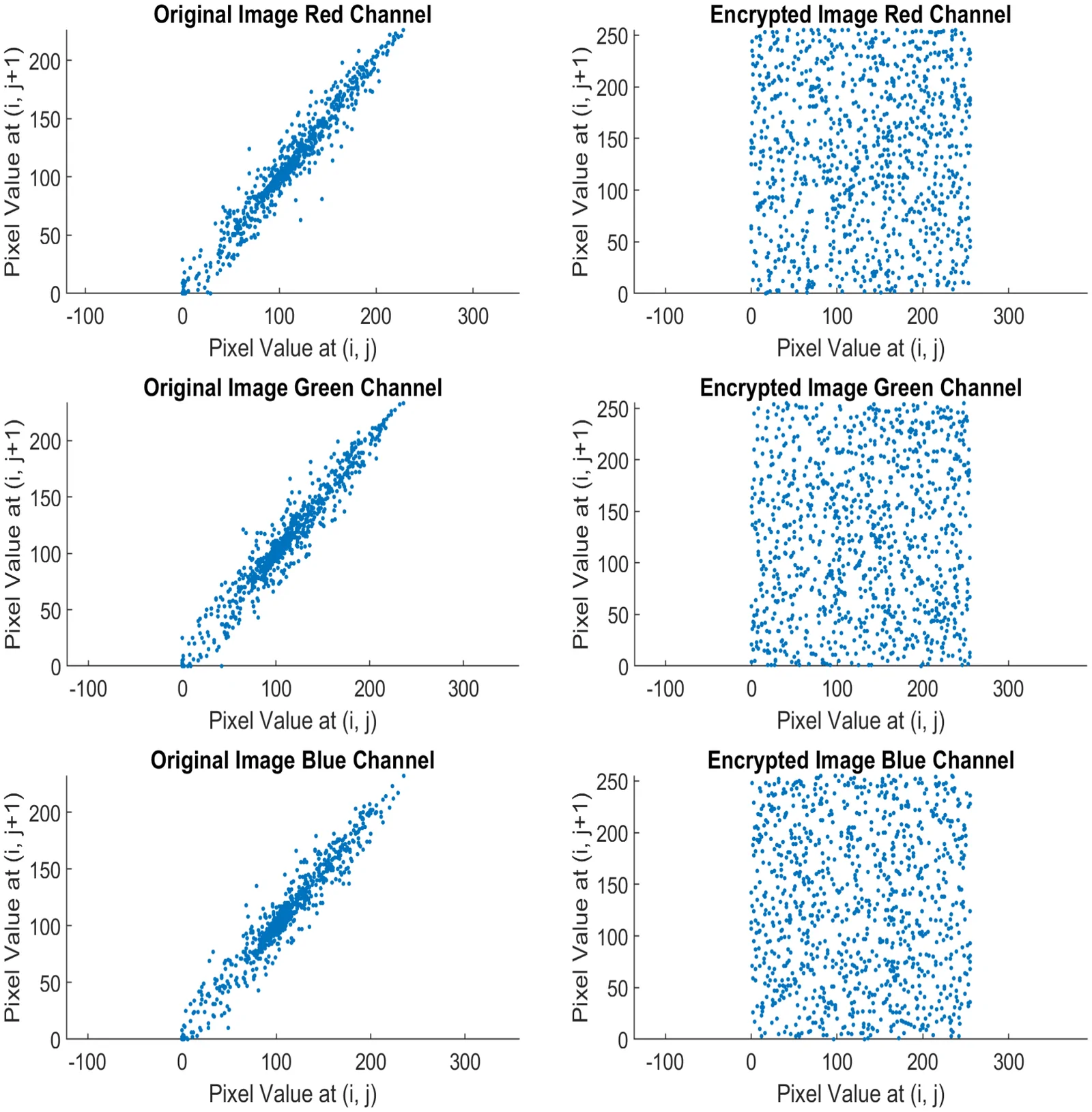
The pixels correlation coefficient of original and encrypted image for Male image.


In image encryption, attacks based on statistics are the most successful methods. The ciphered image must resist statistical attacks by utilizing a secure method. The correlation among the directions of vertical, horizontal, and diagonal has to be analysed for neighbouring pixels in the original and ciphered images [52]. Equations (12), (13), (14), and (15) are utilized to calculate the correlation of image pixels, to verify the ability and the robustness against statistical correlation attacks for the proposed scheme. Figs 10–13 presents the correlation coefficient among neighbouring pixels in an image for horizontal, vertical, and diagonal pixels of original images and encrypted images for different sizes and images.

$$\begin{array}{c} Cor=\frac{cov(x,y)}{\sqrt{D(x)}\sqrt{D(y)}} \end{array}$$ (12)

Where


$$\begin{array}{c} D(x)=\frac{\text{1}}{N}\sum_{i=\text{1}}^{N}{\left(x_{i}-x^{\prime }\right)}^{\text{2}} \end{array}$$ (13)

$$\begin{array}{c} D(y)=\frac{\text{1}}{N}\sum_{i=\text{1}}^{N}{\left(y_{i}-y^{\prime }\right)}^{\text{2}} \end{array}$$ (14)

$$\begin{array}{c} ov(x,y)=\frac{\text{1}}{N}\sum_{i=\text{1}}^{N}\left(x_{i}-x^{\prime }\right)\left(y_{i}-y^{\prime }\right) \end{array}$$ (15)

Where *y* and *x* represent the pairs of *N* neighbouring pixels, D(x), D(y) is the variance, and x′, y′ is the expectations.

### 7.5 Correlation coefficient (CC) among plain and cipher images

The responsibility of the (CC), is to calculate the correlation among original images and associated encrypted images. Equations (16), (17), and (18), are used to calculate the correlation coefficient.


$$\begin{array}{c} \overset{―}{A}=\frac{\text{1}}{M\;\times N}\sum_{i=\text{1}}^{M}\sum_{j=\text{1}}^{N}A_{ij} \end{array}$$ (16)

$$\begin{array}{c} \overset{―}{B}=\frac{\text{1}}{M\;\times N}\sum_{i=\text{1}}^{M}\sum_{j=\text{1}}^{N}B_{ij} \end{array}$$ (17)

$$\begin{array}{c} \;CC=\sum_{i=\text{1}}^{M}\sum_{j=\text{1}}^{N}\left(A_{ij}-\overset{―}{A}\right)\left(B_{ij}-\overset{―}{B}\right)\;/\sqrt{{\left(\sum_{i=\text{1}}^{M}\sum_{j=\text{1}}^{N}\left(A_{ij}-\overset{―}{A}\right)\right)}^{\text{2}}{\left(\sum_{i=\text{1}}^{M}\sum_{j=\text{1}}^{N}\left(B_{ij}-\overset{―}{B}\right)\right)}^{\text{2}}} \end{array}$$ (18)

Here, A and B, are denoted by the original and ciphered images, respectively the sizes of the original and encrypted images are N * M. The significant difference between original and encrypted images is when the value of CC is close to zero. That indicates the robustness of the cryptosystem. Table 3 illustrates correlation coefficient of closeness pixels of the original and cyphered images for each channel in vertical, horizontal, and diagonal directions. Table 4 illustrates the comparison of the correlation coefficient of the proposed method against the previous methods.

**Table 3:** The pixels correlation coefficient of original and encrypted image channels.

Images Size	Original Image				Encrypted Image		
	Channels	Vertical (V)	Horizontal (H)	Diagonal (D)	Vertical (V)	Horizontal (H)	Diagonal (D)
256 x 256 Peppers 4.2.07	Red(R)	0.9635	0.9663	0.9564	−0.0010	−0.0024	−0.0016
	Green(G)	0.9811	0.9818	0.9687	0.0019	−0.0040	0.0013
	Blue(B)	0.9665	0.9664	0.9478	0.0023	−0.0006	−0.0022
256 x 256 4.1.06	Red	0.9590	0.9361	0.9159	0.0058	0.0057	0.0060
	Green	0.9687	0.9457	0.9318	0.0029	0.0051	0.0063
	Blue	0.9612	0.9406	0.9265	0.0082	0.0001	0.0016
N_52	Grayscale	0.9842	0.9839	0.9690	−0.0036	−0.0035	0.0036
512 x 512 2.1.11	Red(R)	0.9720	0.9675	0.9486	−0.0022	−0.0018	0.0016
	Green(G)	0.9731	0.9679	0.9485	0.0001	−0.0012	0.0011
	Blue(B)	0.9590	0.9533	0.9277	−0.0018	−0.0018	0.0011
512 x 512 4.2.06	Red(R)	0.9590	0.9361	0.9159	0.0058	0.0057	0.0060
	Green(G)	0.9687	0.9457	0.9318	0.0029	0.0051	0.0063
	Blue(B	0.9612	0.9406	0.9265	0.0082	0.0001	0.0016
5.2.08	Grayscale	0.9462	0.9528	0.9116	0.0039	0.0000	0.0030
1024 x 1024 2.2.21	Red(R)	0.9043	0.9097	0.8662	0.0005	0.0010	−0.0004
	Green(G)	0.8379	0.8492	0.7874	0.0006	−0.0002	0.0009
	Blue(B)	0.7299	0.7391	0.6723	−0.0001	−0.0006	−0.0018
5.3.01	Grayscale	0.9774	0.9813	0.9671	−0.0005	−0.0009	0.0001
5.3.02	Grayscale	0.9099	0.9034	0.8590	0.0009	−0.0004	0.0001

**Table 4:** Average correlation coefficient of Peppers image comparison with other methods.

Authors	Original Image			Encrypted Image		
	Horizontal(H)	Vertical(V)	Diagonal(D)	Horizontal(H)	Vertical(V)	Diagonal(D)
Proposed	0.9733	0.9764	0.9577	0.0013	−0.0036	0.0019
[20]	0.9559	0.9793	0.9537	− 0.0006	*−* 0.0010	− 0.00003
[26]	0.9594	0.9667	0.9304	0.0053	0.0044	0.0005
[27]	0.9369	0.9272	0.9637	− 0.0174	− 0.0105	− 0.0241
[34]	0.9559	0.9793	0.9537	0.0043	0.0010	0.0018
[35]	–	–	–	− 0.0011	0.0018	0.0046
[23]	–	–	–	0.0045	− 0.0021	0.0075
[53]	–	–	–	0.0016	0.0009	0.0053

### 7.6 Image histogram analysis

**Fig 14:**
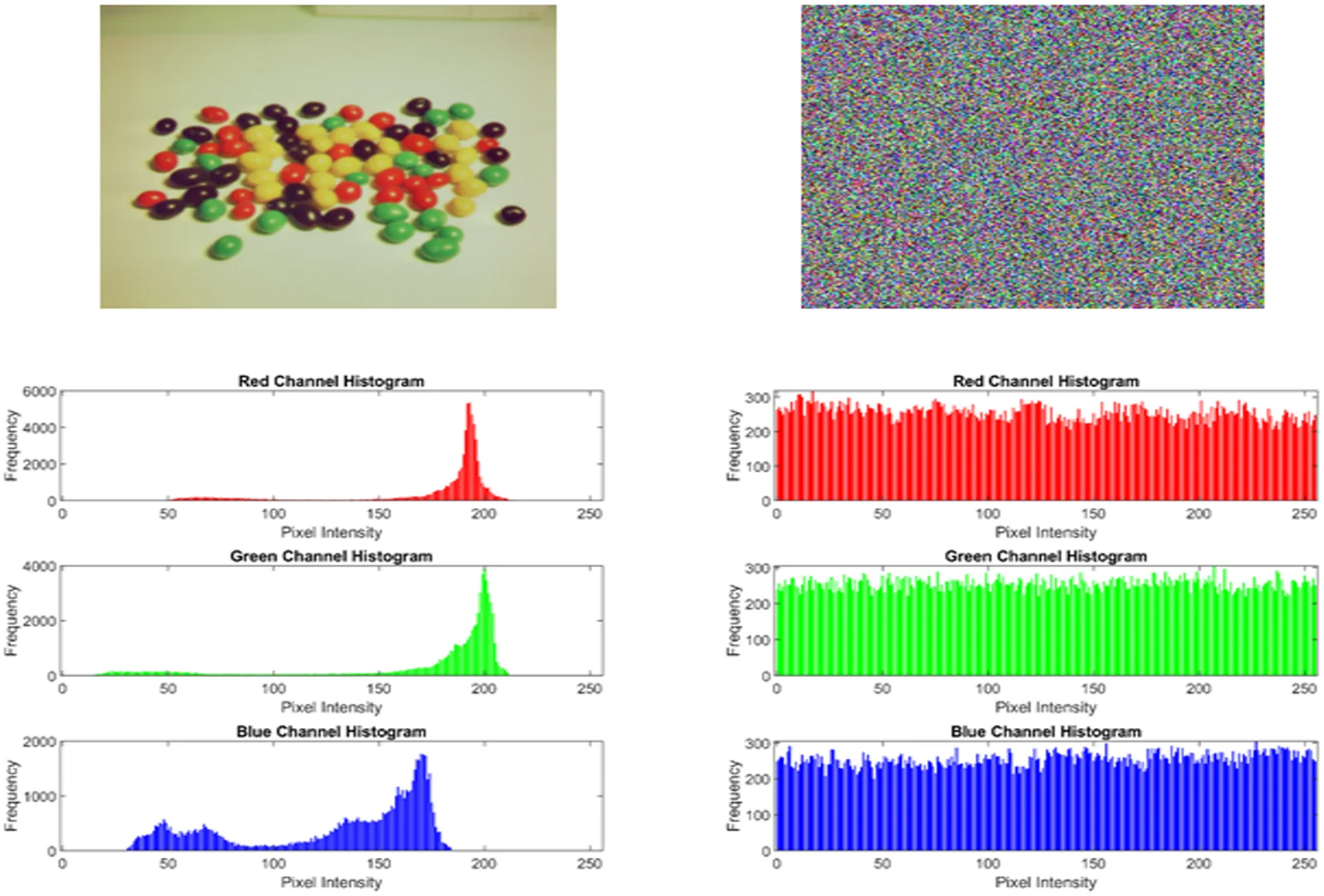
The histogram for the plain image and the ciphered image of the jelly beans image.

**Fig 15:**
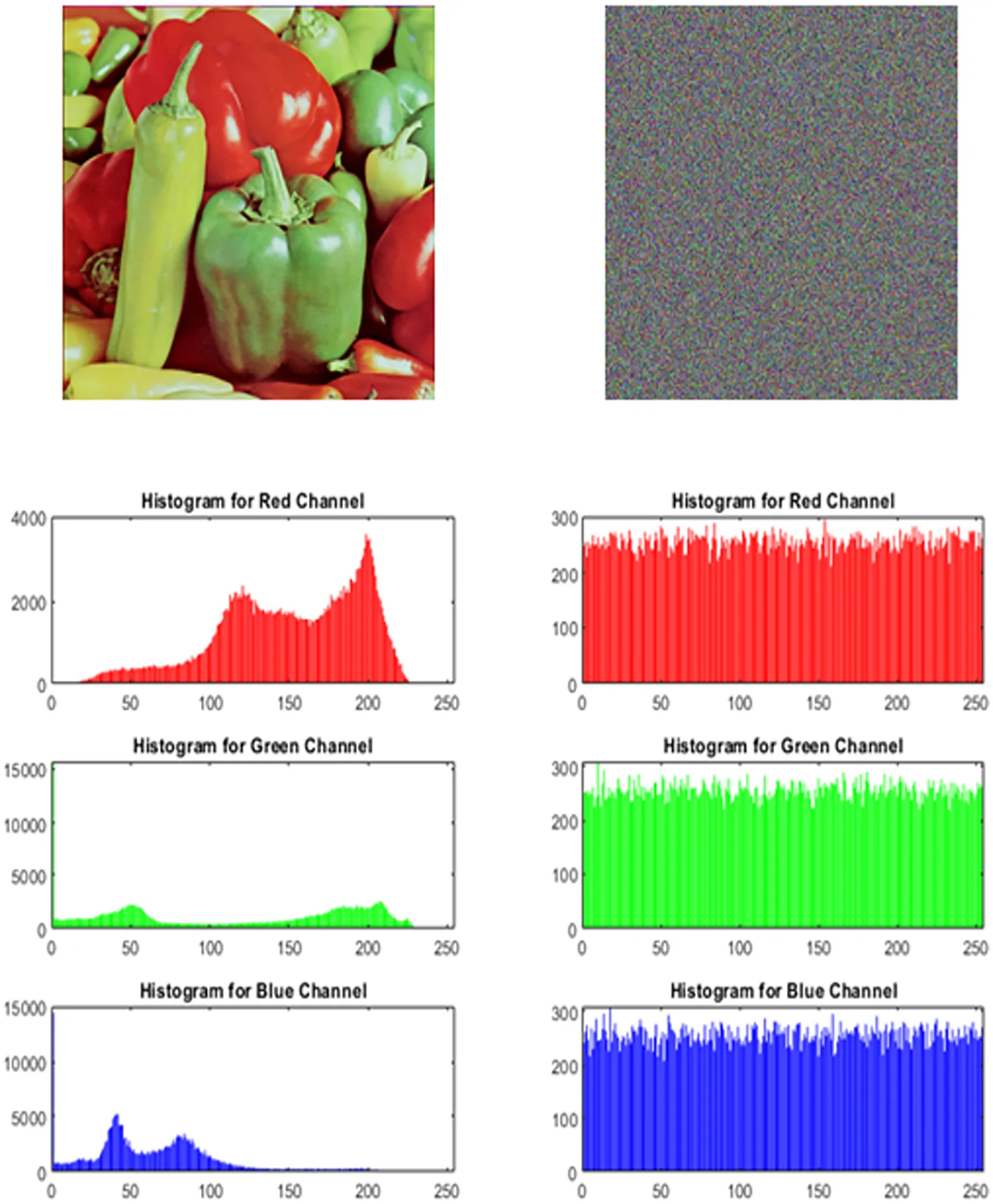
The histogram for the plain and ciphered image of the Peppers image.

**Fig 16:**
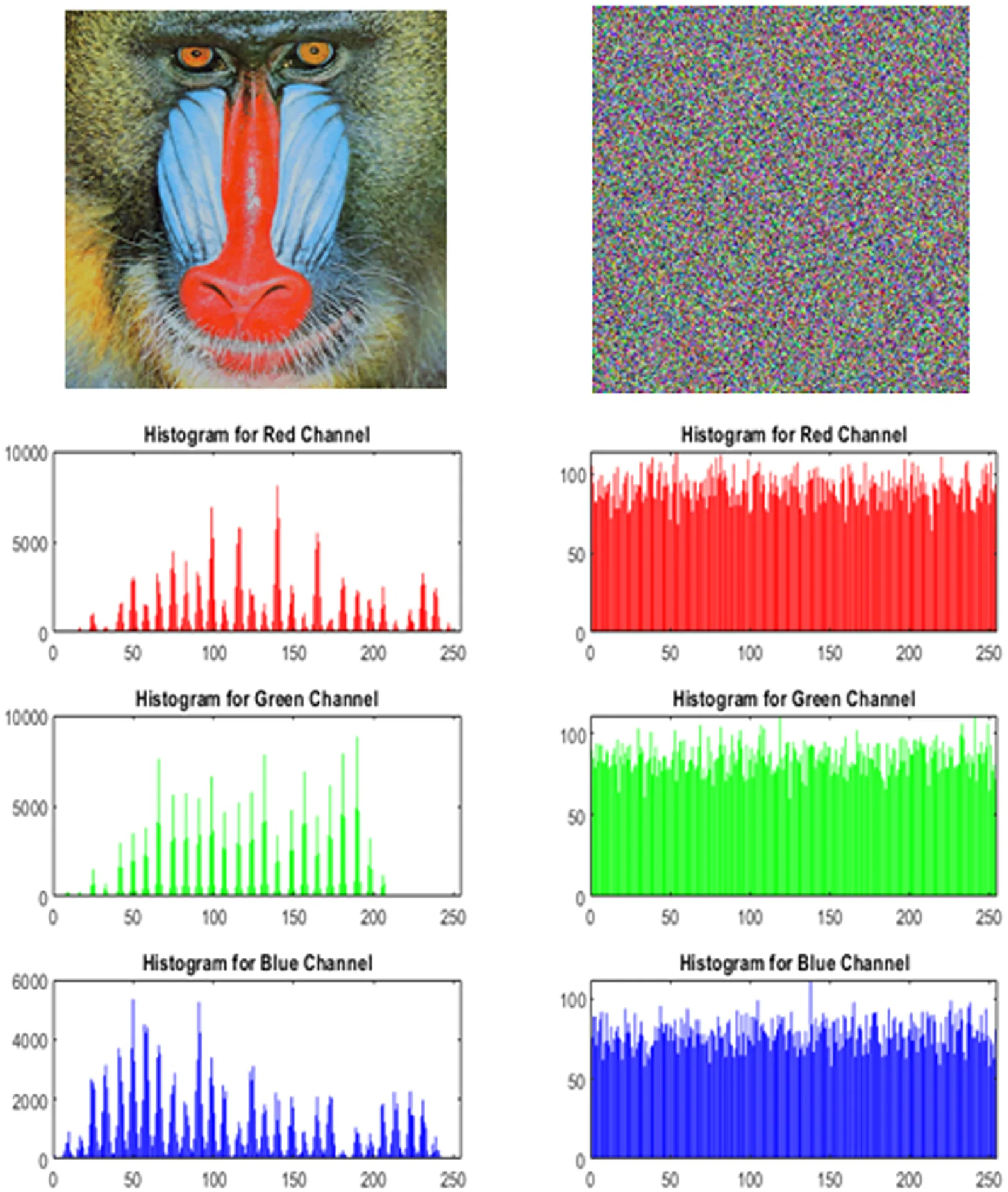
The histogram for the plain and ciphered image of the Mandrill image.

**Fig 17:**
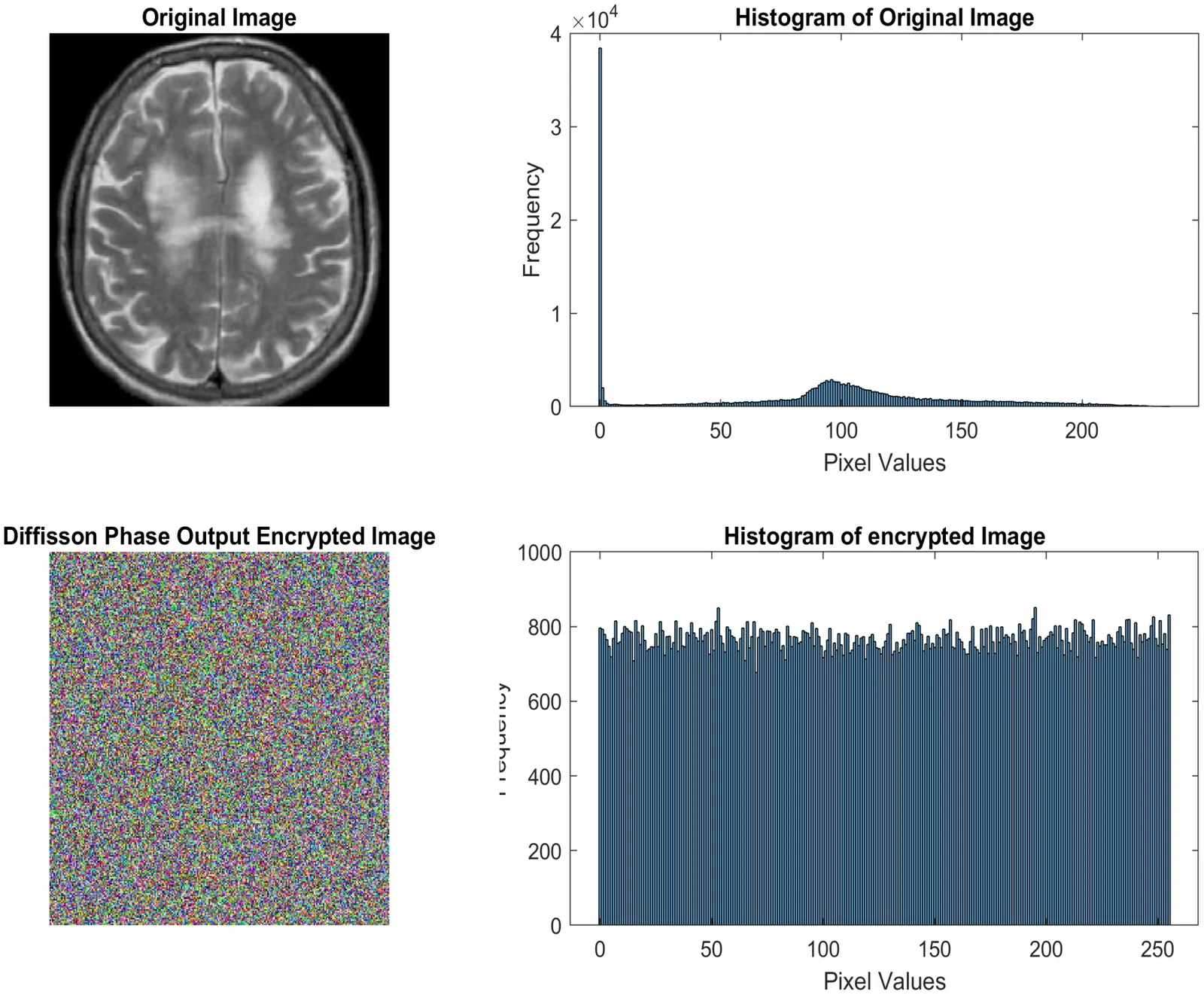
The histogram for the plain image and ciphered image of Medical image of the brain.

**Fig 18:**
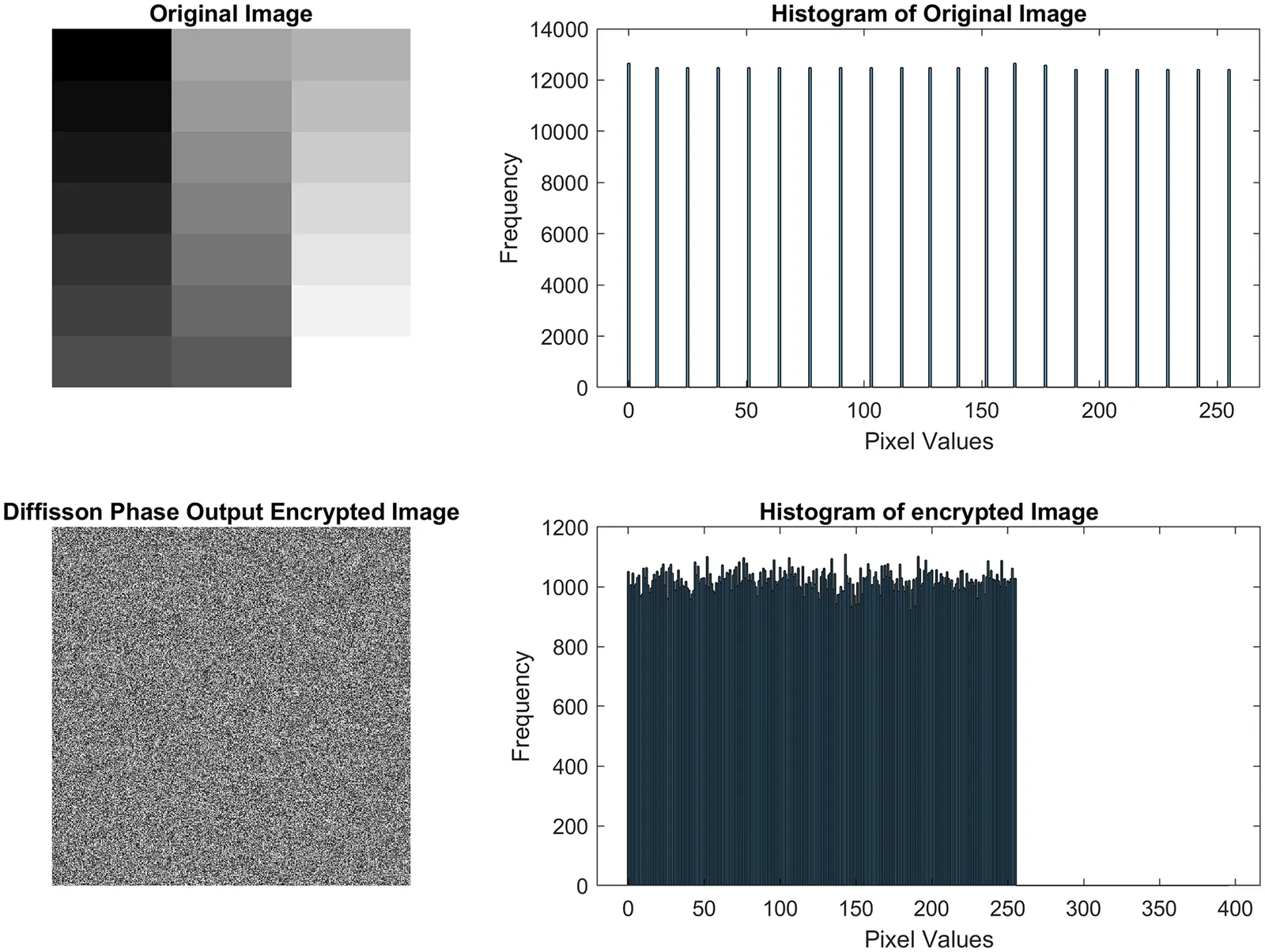
The histogram for the plain and ciphered image of the Gray 21.512 image.

The histogram of an image illustrates the occurrence frequency for every pixel value in an image. To avoid statistical histogram attacks, the ciphered image’s histogram needs to be uniform and statistically distinct from the original image’s histogram [44]. Each bar plot in the image histogram indicates a specified number of appearances for the identical pixel value. The greyscale images (8-bit), pixel values are ordered from 0 (black colour pixel) to 255 (white colour pixel) as presented in Figs 14–18.

If the ciphered image has a flat histogram, it indicates that the histogram has the perfect result as required. This result indicates the sternness of the encryption method in case of statistical attacks. To estimate the value of uniform pixel distribution, it is necessary to utilize quantitative analysis [52]. The quantitative evaluation can be implemented by using the difference metric. The difference in the ciphered image is calculated by equation (19).


$$\begin{array}{c} Var\;(H)=\frac{\text{1}}{n^{\text{2}}}\;\sum_{i=\text{1}}^{n}\sum_{j=\text{1}}^{n}\frac{{\left(H_{i}-H_{j}\right)}^{\text{2}}}{\text{2}} \end{array}$$ (19)

Where H_i_, H_j_ indicates the appearance of various pixel values (i, j).

### 7.7 Information entropy analysis

In a cryptosystem, the uncertain association among random variables is measured based on the information entropy evaluation. The theoretical entropy value of a greyscale image is 8. The ciphered image must have entropy values similar to greyscale values when encryption algorithms are produced [35]. Equation (20) is used to calculate the information entropy.


$$\begin{array}{c} H(m)=\sum_{i=\text{0}}^{M-\text{1}}P(m_{i}){\text{log}}_{\text{2}}\frac{\text{1}}{P(m_{i})} \end{array}$$ (20)

Where M is the total number of pixels, m_i_ represents the probability of the appearance of the character m_i_, and log indicates the base 2 logarithm so that the entropy is shown in binary form. When the encrypted image entropy value is adjacent to 8, that means the encrypted image is approximately haphazard, and the applied algorithm is robust against attacks based on information entropy. Table 5 illustrates the information entropy of images for the proposed method, and Table 6 illustrates the information entropy comparison with other methods.

**Table 5:** The information entropy of images for the proposed method.

File name	Original Image			Encrypted Image		
	Red	Green	Blue	Red	Green	Blue
Peppers 4.2.07	7.3388	7.4962	7.0583	7.9991	7.9992	7.9990
4.1.06	7.2104	7.4136	6.9207	7.9967	7.9972	7.9964
2.1.11	6.5723	7.4511	6.7625	7.9987	7.9982	7.9988
2.1.02	7.4061	7.4188	6.5931	7.9989	7.9992	7.9990
2.2.17	7.2051	6.8229	5.8813	7.9997	7.9998	7.9997
2.2.21	7.3256	6.6328	5.2768	7.9997	7.9998	7.9997
4.2.06	7.7621			7.9997		
Grayscale 5.2.08	7.2952			7.9989		
Grayscale 5.3.01	7.5237			7.9998		
N_52	6.6047			7.9989		
Grayscale 5.3.02	6.8303			7.9998		

**Table 6:** The information entropy of Peppers image comparison with other methods.

Methods	Original image			Encrypted image			Average
	Red	Green	Blue	Red	Green	Blue	
Proposed	7.3388	7.4962	7.0583	7.9991	7.9992	7.9990	**7.9991**
[19]	7.3356	7.6122	7.1604	7.9954	7.9954	7.9955	7.9954
[20]	–	–	–	7.9964	7.9969	7.9969	7.9967
[26]	–	–	–	–	–	–	7.9991
[27]	7.3920	7.3920	7.1738	7.9965	7.9969	7.9932	7.9955
[34]	–	–	–	–	–	–	7.9951
[35]	–	–	–	–	–	–	7.9989
[23]	–	–	–	–	–	–	7.9982
[53]	–	–	–	7.9994	7.9993	7.9993	7.9993

### 7.8 Mean square error (MSE) and peak signal-to-noise ratio analysis (PSNR)

In image encryption, the reliability of the encryption method is evaluated using (MSE) between the original and cipher images. Equation (21) is implemented to achieve the evaluation.


$$\begin{array}{c} MSE=\frac{\text{1}}{M*N}\sum_{i=\text{1}}^{M}\sum_{j=\text{1}}^{N}{\left(a(i,j)-b(i,j)\right)}^{\text{2}} \end{array}$$ (21)

The dimensions of the original and encrypted images are N and M; a(i,j) and b(i,j) are pixel values in the ith row and jth column for the original and encrypted images. Robust image encryption security has a larger MSE value. Moreover, to evaluate the quality of the suggested technique, the PSNR, or peak signal-to-noise ratio, is applied by utilizing equation (22).


$$\begin{array}{c} PSNR=\text{1}\text{0}\;{log}_{\text{1}\text{0}}\frac{{(I}_{max}^{\text{2}})}{MSE} \end{array}$$ (22)

Where Imax represents the maximum value of possible pixels in an image. The evaluation of the imperceptibility factor determines if there is any suspicion of including the secret in the image [35,50]. A successful encryption is when the PSNR value is at a minimum, which indicates significant differences between the original and encrypted images. Table 7 illustrates the MSE and PSNR values of each channel for different images, and Table 8 illustrates the comparison between the proposed method outcome and other methods.

**Table 7:** The MSE and PSNR evaluation.

Image	Channel	MSE	PSNR dB
Peppers 4.2.07	Red	7966.4238	9.1182
	Green	11212.4236	7.6338
	Blue	11056.8093	7.6945
2.1.11	Red	6772.7118	9.8232
	Green	9086.4816	8.5468
	Blue	6677.0986	9.8849
2.2.21	Red	7533.0857	9.3611
	Green	8202.8193	8.9912
	Blue	11098.7045	7.6781
4.1.06	Red	8736.1555	8.7176
	Green	11238.2358	7.6238
	Blue	9786.9551	8.2243
4.2.06	Grayscale	10120.1277	28.6533
5.2.08	Grayscale	7462.8903	9.4017
5.3.01	Grayscale	10338.5616	7.9862
N_52	Grayscale	10532.1503	7.233
5.3.02	Grayscale	8722.4153	8.1978

**Table 8:** Average MSE and PSNR comparison of Peppers image with methods.

Methods	MSE	PSNR dB
Proposed scheme	10078.55	8.1488
[20]	10092.3	8.0908
[26]	10080.2	8.0961
[34]	10298.7	8.0029
[35]	10092.2	8.1181
[23]	9831.3	8.205

### 7.9 Differential analysis

If one minor change in the plain image causes an essential change in the ciphered image, differential analysis will be useless [54]. To verify the security of the encryption method, the difference between the encrypted forms should be as large as possible. If small changes in the original image cause small changes in the encrypted image, such behavior can be exploited by differential analysis by successfully collecting useful information [55]. The Unified Average Changing Intensity (UACI) and the Number of Pixel Change Rate (NPCR) are the most valuable security analyses for differential image encryption attacks. NPCR measures the overall absolute number of changed pixels through a differential attack, while UACI measures the average change in a pair of encrypted pixels. Table 9 illustrates the NPCR and UACI values for different image sizes, Table 10 compares the UACI and NPCR with other methods, and Table 11 illustrates the grayscale image Baboon comparison with other methods.

**Table 10:** The NPCR and UACI comparison of Peppers image with other methods.

Images	Channel	NPCR%	Average	UACI%	Average
Peppers 4.2.07	R(Red)	99.6180	99.6113	33.4689	33.4592
	G(Green)	99.6087		33.4587	
	B(Blue)	99.6073		33.4499	
[19]	R(Red)	99.9500	99.9566	33.2800	33.2900
	G(Green)	99.9600		33.2900	
	B(Blue)	99.9600		33.3000	
[20]	R(Red)	99.6032	99.9536	33.4759	33.4606
	G(Green)	99.6032		33.4702	
	B(Blue)	99.3750		33.4357	
[26]	R(Red)	99.5880	99.6119	28.8918	32.1632
	G(Green)	99.6017		33.8141	
	B(Blue)	99.6460		33.7838	
[27]	R(Red)	99.6275	99.6133	33.2046	33.1437
	G(Green)	99.6174		33.0761	
	B(Blue)	99.6014		33.1504	
[34]	R(Red)	99.6658	99.6348	28.9610	32.1832
	G(Green)	99.6262		33.7841	
	B(Blue)	99.6124		33.8043	
[35]	R(Red)	–	99.5768	–	32.1233
	G(Green)				
	B(Blue)				
[23]	R(Red)	–	99.5948	–	30.3711
	G(Green)				
	B(Blue)				
[53]	R(Red)	99.6030	99.5967	33.2140	33.2250
	G(Green)	99.6250		33.4240	
	B(Blue	99.5650		33.0370	

**Table 11:** Grayscale image baboon comparison with other methods.

Authors	NPCR	UACI	Entropy	CC (H)	CC (V)	CC (D)
Proposed method	99.6013	33.6274	7.9993	0.0008	−0.0017	0.0006
[53]	99.6210	33.4340	7.9993	0.0034	−0.0000	−0.0020
[56]	99.6000	33.4700	7.9993	−0.0003	0.0002	0.0009
[57]	99.7020	33.1340	7.9982	0.0055	−0.0317	0.0017
[58]	99.5800	33.4500	7.6232	−0.0203	0.0038	0.0023
[59]	99.6740	33.5360	7.9991	–	–	–

**Table 9:** The NPCR and UACI evaluation.

Image	Image type	Average NPCR%	Average UACI%
4.2.07	RGB	99.6113	33.4592
2.1.11	RGB	99.6073	33.4921
2.2.21	RGB	99.6073	33.4675
4.1.06	RGB	99.6073	33.4487
4.2.06	RGB	99.6113	33.4732
5.2.08	Grayscale	99.6017	33.4371
5.3.01	Grayscale	99.6072	33.4697
5.3.02	Grayscale	99.6092	33.4644
N_52	Grayscale	99.6085	33.4357

Assume that c1 and c2 are encrypted images before and after small modifications to the original image. The pixel values at grid (i,j) in c1 and c2 are symbolized as c1(i,j) and c2(i,j), and equation (23) defines a 2D array. Equations (24) and (25) describe mathematically the NPCR and UACI, respectively.


$$\begin{array}{c} D(i,j)=\left\{ \begin{array}{c} @r\text{0},\;if\;c\text{1}(i,j)=c\text{2}(i,j)\\ \text{1},\;if\;c\text{1}(i,j)\neq c\text{2}(i,j) \end{array}\right. \end{array}$$ (23)

$$\begin{array}{c} NPCR=\frac{\text{1}}{M*N}\sum_{i,j}^{M,N}D(i,j)*\text{1}\text{0}\text{0}\% \end{array}$$ (24)

$$\begin{array}{c} UACI=\frac{\text{1}}{M*N}\sum_{i,j}^{M,N}\frac{\left|c\text{1}(i,j)-c\text{2}(i,j)\right|}{\text{2}\text{5}\text{5}}*\text{1}\text{0}\text{0}\%\; \end{array}$$ (25)

Where M and N are the dimensions of c1 and c2.

### 7.10 Chosen-plaintext attack analysis

To assess the resilience of the proposed image encryption algorithm against chosen-plaintext attacks (CPA), a controlled experiment was performed in which two plaintext images varying by a single pixel were encrypted using the identical secret key. The resulting ciphertexts were then assessed using two standard sensitivity metrics: the Number of Pixels Change Rate (NPCR) and the Unified Average Changing Intensity (UACI). The experimental results, NPCR = 99.6113% and UACI = 33.6682%, closely approximate the theoretical ideal values of 99.6094% and 33.4635%, respectively. These results confirm that even a minimal alteration in the input leads to a significant and unpredictable change in the ciphertext, thereby indicating strong diffusion characteristics. Such high NPCR and UACI values demonstrate that the proposed scheme exhibits strong resistance to differential and chosen-plaintext attacks, as minor changes in the plaintext cannot reveal any relationship between plaintext–ciphertext pairs. Comparable findings have been reported in recent chaotic encryption studies [55], validating that chaotic dynamics provide excellent sensitivity to initial conditions and plaintext variations, ensuring robust protection against CPA attempts.

### 7.11 Machine learning–based cryptanalysis evaluation

To rigorously assess the robustness of the proposed 4D Lorenz system of image encryption scheme against intelligent attacks, a comprehensive machine learning (ML) cryptanalysis evaluation was conducted in strict accordance with the three-method framework of Singh, Sivangi, and Tentu [60]. represents the current standard for ML-based cryptanalysis evaluation and encompasses three distinct attack categories: stochastic optimisation (2.1), population-based heuristic search (2.2), and deep neural network distinguishing attacks (3/5.4). Each method exploits a different vulnerability surface of a cipher and the three methods are complementary. Failure of all three provides strong multi-layered evidence of security. Standard RGB test images from the USC-SIPI image database are utilized, where each is encrypted using a unique set of Lorenz initial conditions, ensuring fully independent ciphertexts across all experimental trials and eliminating any keystream reuse artifact from the evaluation. All experiments were implemented in MATLAB R2023b with the Deep Learning Toolbox, using a fixed random seed (rng(42)) throughout.

#### 7.11.1 Slippery hill-climbing attack.

**Fig 19:**
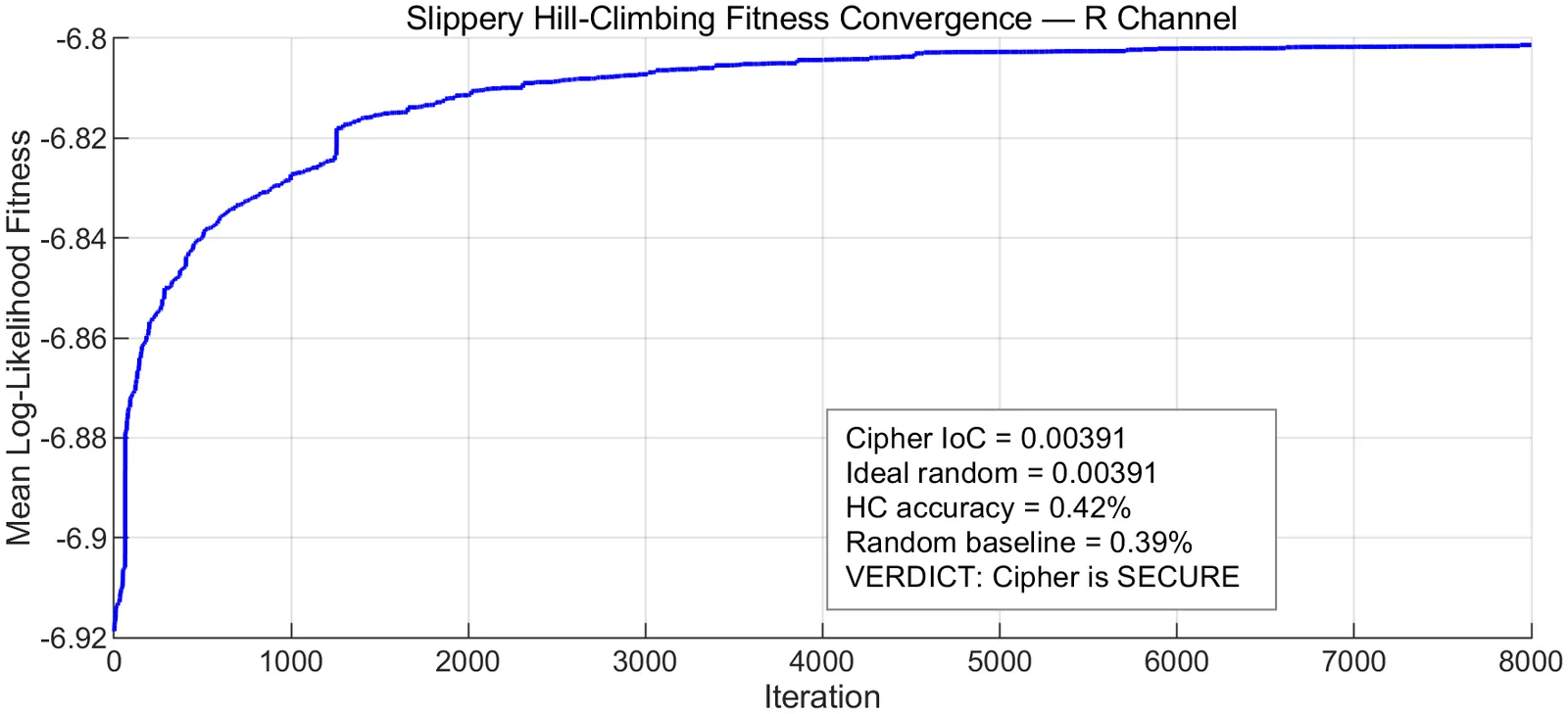
Slippery Hill-Climbing convergence profile across 200 iterations for the representative test image (Image 1, 512 × 512).

The Slippery Hill-Climbing (HC) attack, described by Kaeding [61] and adopted in section 2.1 of [60], is a stochastic ciphertext-only attack that requires no knowledge of the plaintext or encryption key. It attempts to recover the encryption key by searching the key space using guided random exploration. The test was applied to the flattened R-channel byte stream of each cipher image, treating it as a classical substitution ciphertext over a 256-symbol alphabet. The test proceeds in the following steps. In step one, the Index of Coincidence (IoC) of the cipher byte stream is computed as a pre-attack diagnostic, measuring how closely the cipher byte distribution approximates a uniform random distribution (ideal: 1/256 = 0.003906). In step two, a reference byte-frequency distribution is built from the plain image and converted to log-probabilities to form the fitness function. In step three, a parent key is initialised as the identity permutation of the 256-symbol byte alphabet. In step four, a initial key is generated by randomly swapping two elements of the parent key; if the initial key produces a decrypted byte stream with higher log-probability fitness, it replaces the parent. In step five, if no improvement is detected after 300 consecutive iterations, the key is fully re-randomised and the search restarts from a new position. This is the defining ‘slippery’ mechanism that prevents permanent entrapment at local fitness maxima [61]. Steps four and five repeat for 8,000 total iterations per image. In step six, the final key is applied to the cipher, and decryption accuracy is computed as the fraction of pixels correctly recovered. Fig 19 shows the fitness convergence curve of the HC, showing mean log-probability fitness on the y-axis against iteration number on the x-axis. The curve exhibits the characteristic pattern of a fully resistant cipher: an immediate plateau within the first 300 iterations followed by no sustained improvement across all 8,000 iterations. The occasional small upward fluctuations correspond to the slippery re-randomisation steps, each of which restarts the search from a new random position without finding any better local maximum. The annotation box confirms the cipher IoC of 0.003908, identical to the random ideal of 0.003906 to five decimal places. Across all valid images, the HC attack achieved key-recovery accuracies of 0.40–0.42% (mean: 0.41 ± 0.01%), compared to the random-guess baseline of 0.39% (= 1/256). The maximum observed deviation from the baseline was 0.03 percentage points across all test images well within normal random variation. The near-ideal IoC values (0.003906–0.003909 across all images) explain this complete failure with a uniform byte distribution, the fitness function provides no gradient that the algorithm can follow, making the search equivalent to random key guessing. The HC attack was completely ineffective against the proposed cipher.

#### 7.11.2 PSO cryptanalysis.

**Fig 20:**
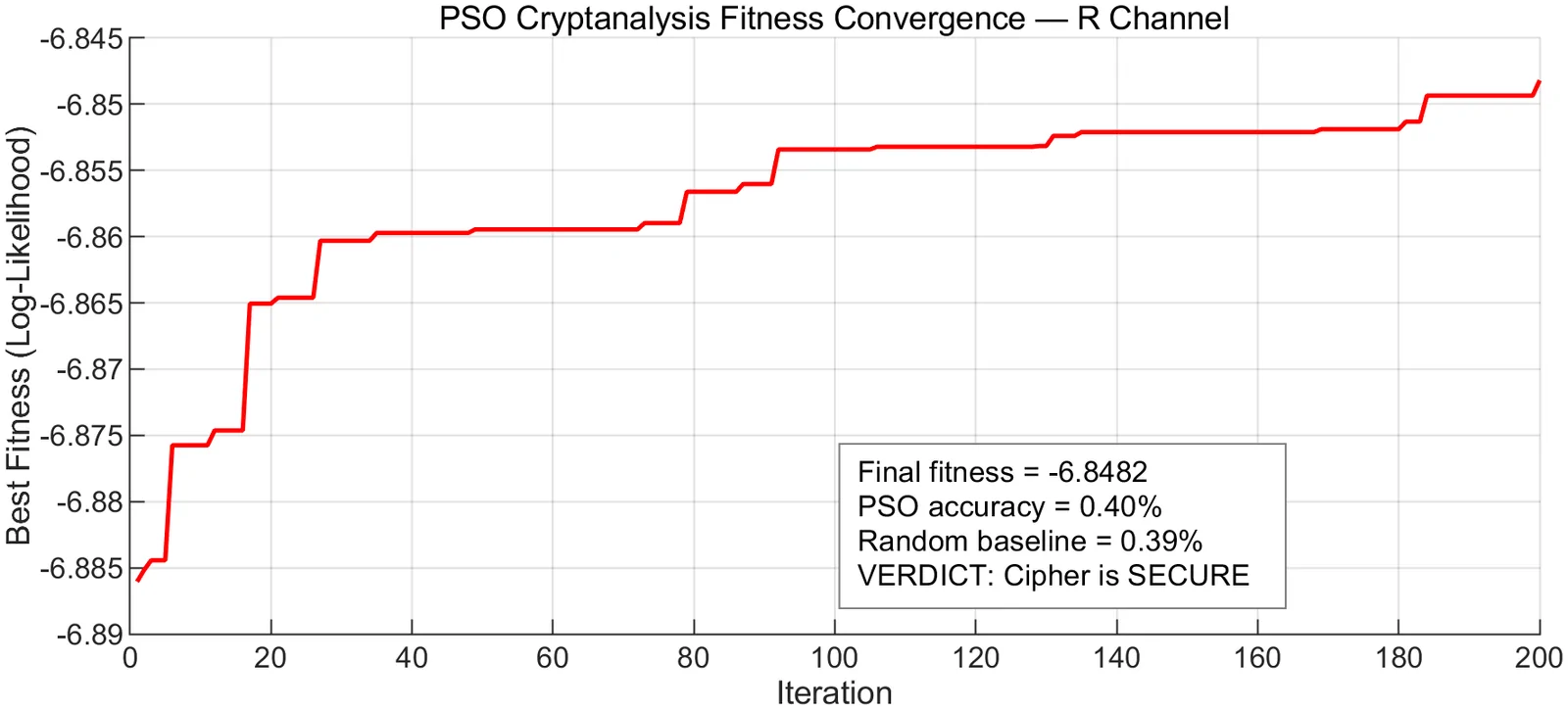
PSO convergence curve across 200 iterations.

The Particle Swarm Optimisation (PSO) cryptanalysis method, formulated by Sadiq et al. [62] and described in Section 2.2 of [60], is a population-based heuristic search that uses collective swarm intelligence to optimise the key search. Unlike HC, which maintains a single candidate key, PSO maintains a swarm of 30 candidate keys simultaneously, allowing it to explore multiple regions of the key space in parallel. The test proceeds as follows. In step one, 30 particles are initialised with random continuous position vectors of dimension 256, each representing a candidate byte-substitution key via its rank-ordering. In step two, each particle’s fitness is evaluated as the mean log-probability of the decrypted byte stream under the plain-image reference distribution. In step three, the global best (gbest) and each particle’s personal best (pbest) are identified. In step four, each particle’s velocity is updated according to Equation 1 of Singh et al. [60]: vᵢ = c₀·vᵢ + c_1_·r_1_·(pbestᵢ − pᵢ) + c_2_·r_2_·(gbest − pᵢ), with inertia weight _0_ = 0.7, cognitive coefficient c_1_ = 1.4, and social coefficient c_2_ = 1.4. In step five, the position of each particle is updated and fixed into a valid byte permutation by putting them in order of rank. In step six, fitness is re-evaluated, and personal and global bests are updated if improved. Steps four through six are repeated for 200 iterations. In step seven, the global best key is applied to the cipher and recovery accuracy is assessed. Fig 20 presents the PSO global best fitness convergence curve across 200 iterations. The curve shows an immediate plateau: the global best fitness reached approximately −6.86 (log-probability units) at iteration 1 and improved by only 0.024 log-probability units across all remaining 199 iterations. This flat convergence profile is the definitive signature of a cipher whose byte distribution is indistinguishable from uniform random; the swarm finds no exploitable fitness gradient to follow and effectively random-walks through the key space. The annotations confirm the final fitness value and the recovery accuracy relative to baseline. Across all the test valid images, the PSO swarm achieved key-recovery accuracies of 0.39–0.41% (mean: 0.40 ± 0.01%), indistinguishable from the random-guess baseline of 0.39%. The strongest individual result was image 4, whose PSO accuracy of 0.39% matched the random baseline exactly and whose IoC of 0.003906 matched the theoretical ideal exactly to six decimal places, the closest observed result to the theoretical optimum across the entire evaluation. The PSO attack failed completely against all five images.

#### 7.11.3 DNN neural distinguisher.

**Fig 21:**
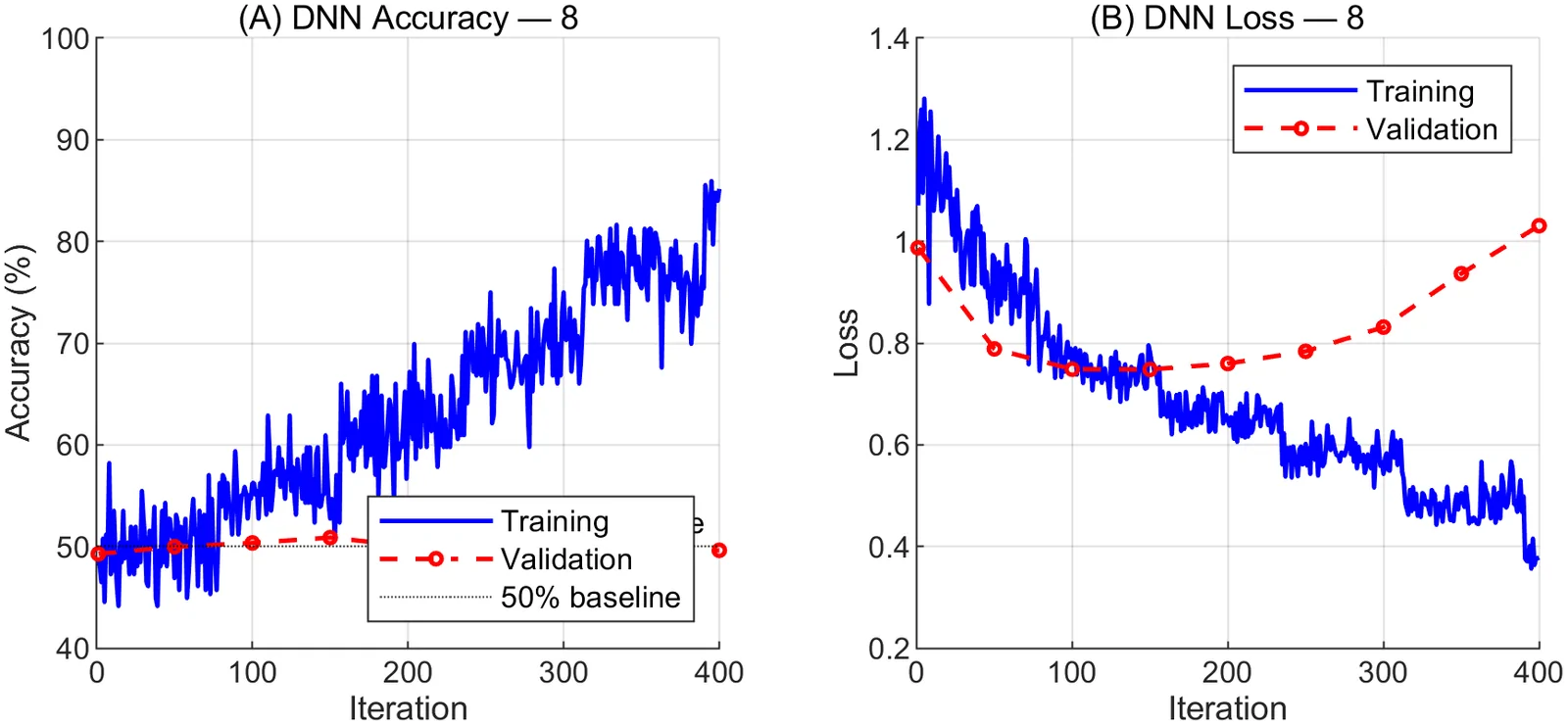
DNN training and validation accuracy and loss curves for Image (1024 × 1024).

**Fig 22:**
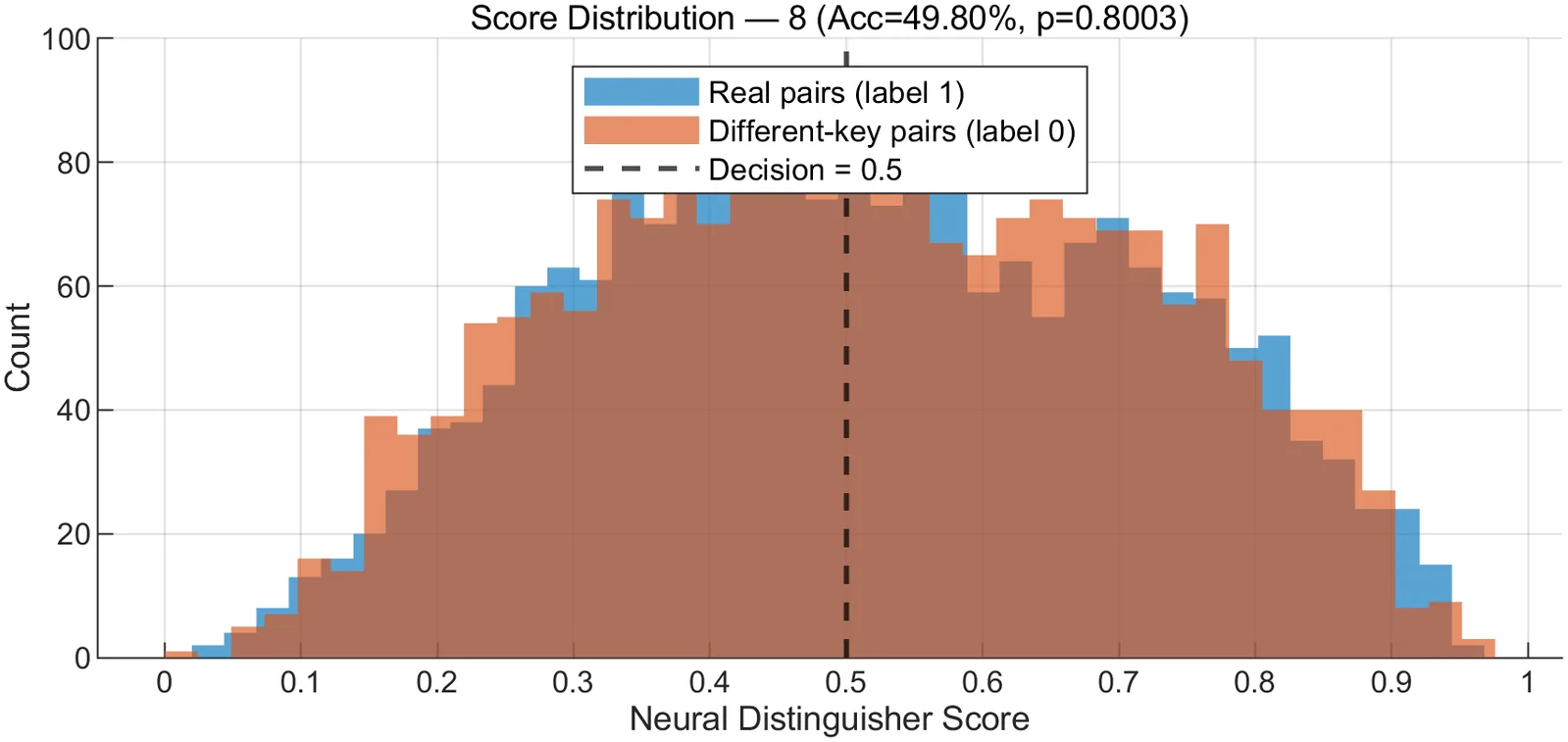
Score distribution histogram produced by the DNN distinguisher for Image (1024 × 1024).

**Fig 23:**
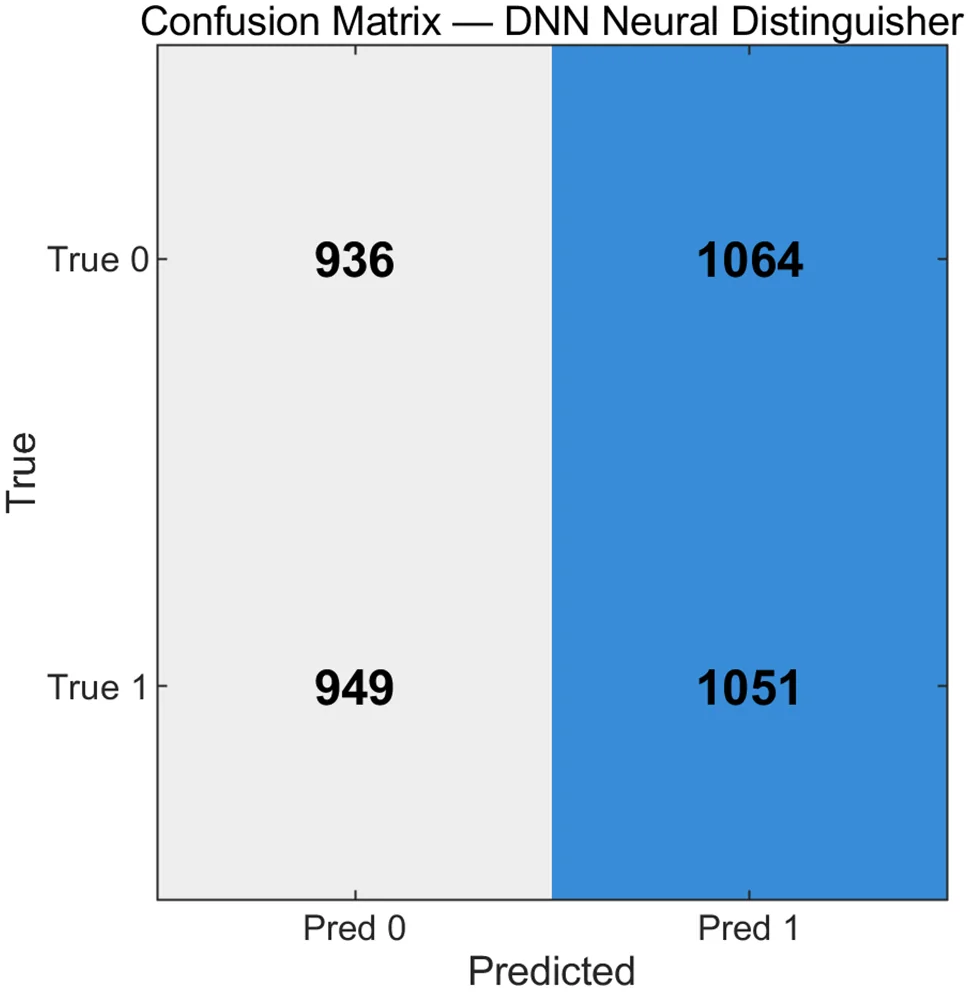
Confusion matrix for the DNN distinguisher across the test set.

The network architecture follows the Gohr [63] ResNet design that is mentioned in section (5.4 of [60]). Block 1 is the initial convolutional block: a one-dimensional convolutional layer with kernel size 1 and 32 filters, followed by batch normalisation and a ReLU activation function, which performs a non-linear mixing of the input bit features. Block 2 consists of two residual units, each containing two one-dimensional convolutional layers with kernel size 3 and 32 filters, each followed by batch normalisation and ReLU, which extract higher-order spatial patterns from the feature representation. Block 3 is the classification head: two fully connected layers of 64 and 32 units with batch normalisation, ReLU, and dropout regularisation at rate 0.5, followed by a two-class softmax output assigning probabilities to the ‘real pair’ and ‘random pair’ classes. Training used the Adam optimiser with an initial learning rate of 1 × 10^-3^, reduced by a factor of 0.1 at epoch 15, and L2 weight decay of 1 × 10^-3^, over a maximum of 25 epochs with a mini-batch size of 256. The training and validation datasets were constructed independently for each test image as follows. In step one, the cipher image is converted to a grayscale representation by averaging the three RGB channels. In step two, label-1 (real) pairs are created: two 16 × 16-pixel patches are extracted from two different spatial locations within the same cipher image, preserving the original spatial ordering of pixels. These represent genuine cipher patch pairs from the same encryption. In step three, label-0 (random) pairs are created: one patch from the cipher image is paired with a patch from a second independently generated ciphertext produced by applying a deterministic perturbation stream to the original ciphertext to simulate a different encryption key. This construction ensures both label classes share identical marginal pixel distributions, so the network must detect spatial structure differences rather than distribution differences, a critical requirement for a valid ND test [60,63]. In step four, each patch is converted to a 2,048-bit binary vector (16 × 16 × 8 bits), and two patches are concatenated to give a 4,096-bit input feature vector. In step five, 20,000 training pairs and 4,000 validation pairs are generated in a class-balanced format. An early-stopping criterion (Validation Patience = 5) terminates training when no validation improvement is detected across five consecutive checks**.** Fig 21 presents the DNN training curves for image 8, the image with the strongest security result. Panel A shows training accuracy (blue line) rising progressively from approximately 48% to 85% across epochs 1–6, while validation accuracy (red dashed line) remains completely flat at approximately 49.5–50.5% throughout, never departing from the 50% random baseline (dotted line). Panel B shows the corresponding loss curves: training loss steadily decreasing from approximately 1.07 to 0.38, while validation loss remains flat and begins to slowly increase as training loss falls. This pattern of training loss decreasing while validation loss stays flat or rises is the characteristic signature of a network that memorises the training data without learning any generalised patterns. At epoch 6, the early stopping criterion correctly terminated training when no validation improvement was detected, preventing unnecessary computation. This convergence pattern was consistent across all the test images, with early stopping triggering at epoch 5 for all images. Fig 22 illustrates the Neural Distinguisher score distribution for the image. The x-axis shows the ND score (probability of being a real pair, ranging from 0 to 1), and the y-axis shows the count of validation samples. The blue histogram shows the score distribution for real pairs (label 1), and the orange histogram shows the distribution for random pairs (label 0). Both distributions overlap almost completely and are centred on the 0.5 decision boundary (dashed vertical line), with no separation between classes. A well-performing distinguisher would show blue scores concentrated above 0.5 and orange scores concentrated below 0.5; the complete overlap observed here confirms that the network assigns nearly random scores to both classes, consistent with inability to distinguish between real and random cipher patches. Fig 23 presents the confusion matrix for the DNN classification. The matrix shows near-equal counts in all four cells: true negatives (TN), false positives (FP), false negatives (FN), and true positives (TP) are all approximately equal, reflecting a classifier that is essentially guessing randomly. A successful distinguisher would show high TN and TP counts with low FP and FN; the balanced confusion matrix observed here is fully consistent with 50% accuracy.

#### 7.11.4 Statistical significance testing.

A two-tailed binomial z-test was used on each DNN result to provide formal inferential confirmation beyond the descriptive accuracy values. The null hypothesis was H₀: accuracy = 50% (cipher indistinguishable from random), and the significance level was α = 0.05. A p-value above 0.05 means the null hypothesis cannot be rejected; the accuracy is not significantly different from 50%, formally confirming cipher security. A p-value below 0.05 means the accuracy is statistically distinguishable from 50%, warranting investigation. All the images produced p-values ranging from 0.137 to 0.800, all substantially above 0.05, formally confirming that their DNN accuracies are not significantly different from random chance at the 95% confidence level. The complete results for some sample images used in the test are summarised in Table 12. Across all three attack methods and all test images, the proposed 4D Lorenz chaos-based encryption scheme demonstrated complete resistance to ML-based cryptanalysis.

**Table 12:** Results across sample of test images across all three attack methods.

Image	HC Acc.	PSO Acc.	DNN Val. Acc.	F1 Score	z-stat.	p-value	Verdict
Image 1	0.42%	0.40%	49.68%	51.08%	−0.411	0.681	SECURE
Image 2	0.41%	0.41%	50.50%	52.54%	0.633	0.527	SECURE
Image 3	0.41%	0.41%	51.18%	52.97%	1.486	0.137	SECURE
Image 4	0.40%	0.39%	49.80%	50.61%	−0.253	0.800	SECURE
**Mean±SD**	**0.41 ± 0.01%**	**0.40 ± 0.01%**	**50.29 ± 1.01%**	**51.80 ± 0.99%**	**0.364**	**—**	**SECURE**
**Ideal**	**0.39%**	**0.39%**	**50.00%**	**—**	**—**	**>0.05**	**—**

### 7.12 Synchronization attack resistance

**Fig 24:**
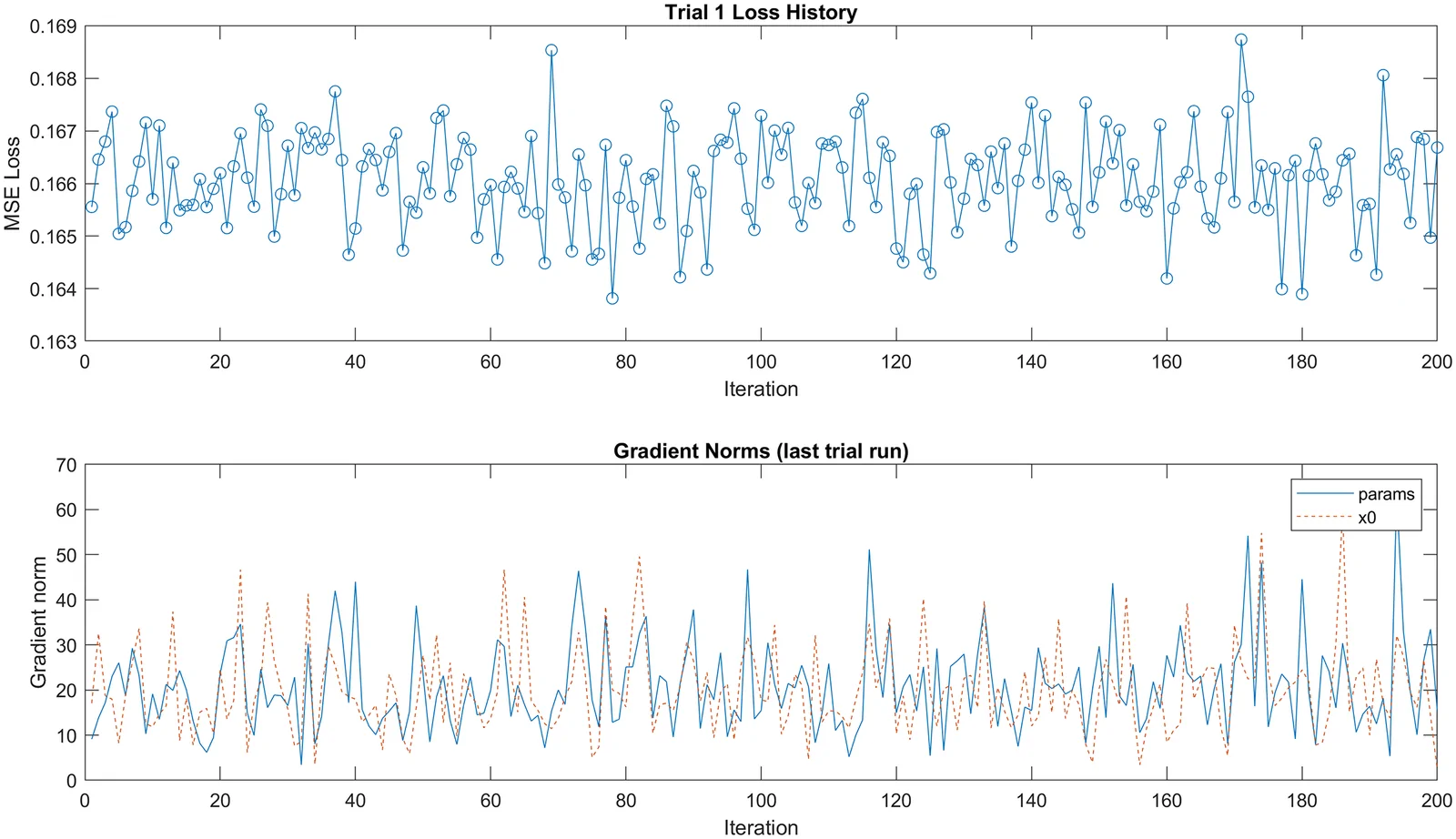
Synchronization attack resistance loss curve.

We assessed the resilience of the proposed image encryption technique against chaotic synchronization-based assaults using an adaptive reconstruction experiment that utilizes the Adam optimization algorithm. In this experiment, an attacker attempts to align the chaotic system parameters of the encryption model by reducing the mean square error (MSE) between the produced and intended ciphertexts. Fig 24 demonstrates that the loss curve displays persistent oscillations around an average MSE of around 0.165, with no discernible convergence after 200 iterations. Simultaneously, the gradient norm exhibits erratic variations, indicating inconsistent update trajectories and unstable learning dynamics. The results demonstrate that the attacker’s model fails to synchronize with authentic chaotic dynamics, since minor changes in the parameters provide markedly diverse paths inside the hyperchaotic state space.

This instability illustrates the intrinsic resistance of hyperchaotic systems to synchronization attacks, which is attributable to their pronounced sensitivity to beginning conditions and high-dimensional coupling [42].

## 8 Comparison with conventional baseline methods

To further demonstrate the effectiveness of the proposed encryption technique, it was compared with three conventional baseline approaches:

(1) Shift-based permutation (position-only scrambling),
(2) XOR-only encryption (bitwise diffusion without permutation), and
(3) Mod-based substitution (intensity alteration without positional change).

All experiments were performed under the same simulation environment using a 256 × 256 grayscale test image.

(a) **Shift-Based Permutation**
In the baseline, each pixel row and column was cyclically shifted according to pseudo-random values derived from a secret key. Only pixel positions were altered, while pixel intensities remained unchanged. The encrypted image showed visual disorder, but the histogram remained non-uniform, and the correlation among adjacent pixels remained relatively high, confirming the limited diffusion capability of permutation-only encryption.
(b) **XOR Encryption**
In XOR-based encryption, pixel values are bitwise XORed with a pseudo-random key stream, providing value alteration but no positional permutation. Although this method achieved a high histogram uniformity, it exhibited weak resistance to differential attacks because identical positional relationships were preserved across the image.
(c) **Mod-Based Substitution**
In mod-based substitution, the encryption is achieved by adding a key stream to each pixel intensity under modulo-256 arithmetic. This modifies pixel values while maintaining their positions. Although this method achieved a high histogram uniformity, it exhibited weak resistance to differential attacks because identical positional relationships were preserved across the image.
(d) **Comparative Results and Discussion**
The performance of all baseline methods was quantitatively assessed using standard evaluation metrics: information entropy, correlation coefficients in three directions (horizontal, vertical, and diagonal), and differential attack resistance measures (NPCR and UACI). The outcomes are summarized in Table 13.

**Table 13:** Comparative Results for the conventional methods with the proposed method.

Encryption Method	CC (H)	CC (V)	CC (D)	NPCR%	UACI%	Entropy
Original image	0.9733	0.9764	0.9677	0	0	7.2977
Shift-Based Permutation	0.0507	0.0650	− 0.0009	99.6086	29.9201	7.9967
XOR-Only Encryption	−0.0025	−0.0046	− 0.0026	96.5954	26.4981	7.9751
Mod-Based Substitution	0.0361	0.0396	0.0364	99.8161	29.3793	7.9784
**Proposed method**	**0.0013**	**−0.0036**	**0.0019**	**99.6113**	**33.4592**	**7.9991**

The results indicate that the proposed algorithm achieves near-ideal entropy and extremely low pixel correlation while maintaining high NPCR and UACI values that are close to theoretical expectations for a perfectly random cipher image. In contrast, the shift-only scheme failed to achieve sufficient diffusion, while XOR-only or mod-based methods lacked adequate spatial confusion. These comparisons show that the proposed hyperchaotic system’s combination of strong permutation and diffusion stages greatly improves encryption performance and security. The comparative study validates that single-operation encryption techniques, whether based solely on permutation, XOR, or modular substitution, are insufficient to ensure complete image confidentiality. Only the combination of nonlinear permutation and diffusion, as employed in the proposed scheme, achieves better resistance to statistical, differential, and plaintext attacks. This confirms the superiority and balanced design of the developed encryption algorithm.

## 9 Computational efficiency measurements

The suggested encryption technique exhibits competitive computing performance when evaluated against current chaotic image encryption methods documented in recent literature. The performance evaluation was executed on a standard computer platform with an Intel® Core™ i3-5010U CPU (2.1 GHz) and 8 GB of RAM, utilising MATLAB R2023 for implementation. The technique encrypts a 512 × 512 colour image (about 0.75 MB) in 0.798 seconds, yielding a throughput of 3.76 MB/s (962.59 KB/s) and processing around 246,424 pixels per second. These metrics are advantageous when compared to recent implementations: a secure image encryption scheme employing a 5D hyperchaotic system in conjunction with Arnold’s Cat Map reported encryption times of 0.1602 seconds for 256 × 256 images [64], whereas a biomedical image encryption scheme using hyperchaotic logistic maps achieved approximately 0.3 seconds for 512 × 512 images [65]. However, direct comparison necessitates accounting for varying hardware configurations and programming environments.

A comprehensive temporal study indicates that the development of the 4D hyperchaotic Lorenz system necessitates 0.080 seconds (10.05% of total execution time), showing effective chaotic sequence generation on minimal hardware. This performance is analogous to lightweight Lorenz-based systems that employ bit-wise XOR operations with pseudo-random sequences [66]. The amalgamation of hyperchaotic Lorenz systems with DNA encoding methodologies underscores the adaptability of this chaotic system in modern encryption frameworks, however such hybrid strategies often entail increased processing demands. The generation of keys from chaotic sequences takes 0.215 seconds (26.99%), constituting the algorithm’s main computational bottleneck on the dual-core i3 processor. This phenomenon is similarly noted in cloud-based chaos encryption implementations, where key generation and pre-processing operations account for substantial portions of execution time [67]. The fundamental cryptographic procedures exhibit exceptional efficiency on mid-range hardware: the confusion phase concludes in 0.016 seconds (1.95%), while the diffusion phase necessitates 0.029 seconds (3.65%), together comprising just 5.6% of the overall execution time.

The memory use for encryption amounts to 34.38 MB, comfortably fitting inside the 8 GB RAM limit, which includes the original image, encrypted output, four chaotic sequences, and intermediate processing arrays. This moderate memory footprint guarantees scalability for bigger images on the identical hardware platform. The throughput of 3.76 MB/s on the i3-5010U processor situates this implementation competitively within the moderate-to-high efficiency spectrum for software-based chaotic encryption, especially in comparison to elliptic curve cryptography methods that incorporate chaotic systems and genetic algorithms for improved security [68]. Specific parallel processing implementations, like the multiple colour image encryption method, achieve 96.4 Mbps throughput and provide enhanced performance via hardware optimisation and parallel execution. Conversely, the proposed software implementation on standard computing platforms exhibits practical feasibility for secure image storage, medical imaging archival, and confidential document transmission applications, where sub-second latency and standard hardware compatibility are acceptable. The algorithm’s performance on the dual-core i3 CPU indicates possibilities for improved throughput on contemporary multicore computers or when optimised using parallel computing frameworks. The equilibrium between security, offered by the 4D hyperchaotic Lorenz system with its extensive parameter space and intricate attractors, and computing efficiency on conventional hardware renders this approach competitive in current chaotic image cryptography studies. Comprehensive evaluations of chaos-based image encryption indicate that contemporary systems must overcome the shortcomings of conventional encryption techniques, which face challenges related to high pixel correlation and image redundancy, while ensuring accessibility for implementation across various computer environments.

## 10 Execution time analysis

The complexity and performance of the proposed algorithm are assessed according to execution time to ensure that the cryptosystem is suitable for application purposes in real time. Table 14 represents the comparison of encryption runtime for the proposed method with other methods in terms of machine specs. We note that the proposed algorithm achieved a suitable runtime for encryption. In addition, the execution time relies on many factors, including the specifications of the machine utilized to run the algorithm, the software implementing the algorithm, and the algorithm itself.

**Table 14:** Execution time comparison of the proposed algorithm and previous methods on the Lena image from dataset USC-SIPI.

Algorithm	Time (s)	Machine specs. (CPU and RAM)
Proposed	0.798	Intel® Core™ i3, 5010U(2.1 GHz), 8 GB RAM
[20]	2.5823	Intel® Core™ i9 (2.9 GHz), 32 GB RAM.
[26]	0.9606	Intel® Core™ i9 (2.9 GHz), 32 GB RAM.
[27]	19.922	Intel® Core™ i3 (1.7 GHz), 4 GB RAM.
[34]	6.057	Intel® Core™ i9 (2.9 GHz), 32 GB RAM.
[35]	0.0216	3.3GHz AMD^®^ Ryzen9 590HX, 32 GB RAM

## 11 Conclusion

This paper introduces a novel image encryption manner based on the hyperchaotic Lorenz system and polar-to-Cartesian coordinate transformation equations. Confusion and diffusion for high cryptosystem security are implemented based on Shannon’s ideas. Although further optimization of the proposed scheme could marginally improve statistical measures such as UACI, NPCR, or entropy, such adjustments would inevitably increase execution time and computational complexity. The primary contribution of this work is therefore not limited to maximizing statistical values but lies in introducing a novel encryption framework that combines complex hyperchaotic equations with bit-level permutation in a manner that is both efficient and practical. This balance between achieving strong cryptographic performance and maintaining simplicity of implementation ensures that the proposed algorithm is well-suited for real-time and resource-constrained applications, where both security and speed are equally critical. The performance of the proposed algorithm was assessed according to various metrics, including an information entropy correlation coefficient analysis, MSE, PSNR, histogram analysis, differential attack (NPCR, UACI), key space, and execution time analysis. The proposed algorithm’s outcome showcases the ability to resist various cryptanalytic attacks and statistical, differential, and brute-force attacks. Finally, the result values of the several metrics of the proposed algorithm present an equivalent or superior achievement compared to other methods from literature, especially the correlation coefficients for the ciphered images. Furthermore, by using multi-chaotic maps, our proposed scheme can extend to other media types, such as text, audio, and video, or be combined with multimedia images in one encryption algorithm.
